# Restoring *Muribaculum intestinale*–Derived Butyrate Mitigates Skeletal Muscle Loss in Cancer Cachexia

**DOI:** 10.1002/jcsm.70140

**Published:** 2025-11-26

**Authors:** Li Li, Panpan Lian, Wei Dong, Shiyu Song, Junaid Wazir, Ranran Wang, Kai Lin, Wenyuan Pu, Renwei Lu, Zhenghong Yu, Chao Ding, Zhiqiang Huang, Yong Wang, Hongwei Wang

**Affiliations:** ^1^ State Key Laboratory of Analytical Chemistry for Life Science, Medical School Nanjing University Nanjing P. R. China; ^2^ Center for Translational Medicine and Jiangsu Key Laboratory of Molecular Medicine, Medical School Nanjing University Nanjing P. R. China; ^3^ Nanjing Lupine (YuShanDou) Biomedical Research Institute Co., Ltd. Nanjing P. R. China; ^4^ Department of Rheumatology and Immunology, Jinling Hospital, Affiliated Hospital of Medical School Nanjing University Nanjing China; ^5^ Department of General Surgery, Nanjing Drum Tower Hospital, Affiliated Hospital of Medical School Nanjing University Nanjing China

**Keywords:** cancer cachexia, gut microbiota, *Muribaculaceae*, *Muribaculum intestinale*, muscle atrophy, short‐chain fatty acid

## Abstract

**Background:**

Muscle wasting in cancer cachexia patients is a major clinical challenge. Although reduced levels of short‐chain fatty acids (SCFAs) in cachexia patients have been associated with muscle atrophy, their precise role remains unclear. Given that the gut microbiota is the primary source of SCFAs, modulating SCFA composition through probiotic supplementation has shown promise in preclinical studies of cancer cachexia. In this study, we aimed to elucidate the dysregulation of the gut microbiota in cachexia mice and investigate the potential protective effect of supplementation with the inulin diet, *Muribaculum intestinale* (MI) and sodium butyrate (NaB) against cachexia‐induced muscle wasting.

**Methods:**

We analysed the gut microbiota composition using 16S rRNA gene amplicon sequencing and measured SCFA levels to evaluate metabolic changes in faecal samples from cancer cachexia models. We identified the associations between the microbiota and metabolites and evaluated the impacts of MI (10^8^ CFU per mouse), NaB (50 mg/kg) and inulin diet on cancer cachexia models. The mechanism of NaB was elucidated by muscle RNA‐Seq and confirmed by Western blotting, qPCR, ATP assays and other experimental approaches, revealing the effects of altered gut microbiota composition and metabolite levels on muscle metabolism in cachectic mouse models.

**Results:**

Faecal analysis in cachectic mice revealed a significant alteration in gut microbiota composition, particularly a reduction in *Muribaculaceae* (76.0%) and *Muribaculum intestina*le (82.0%). Direct supplementation with MI increased its abundance and butyrate level (*p* < 0.05), reducing muscle wasting in cachexia. Correlation analysis underscored a significant positive association between *Muribaculaceae*, *Muribaculum intestinale* and butyrate levels (*p* < 0.05). NaB also ameliorated muscle wasting, with RNA‐Seq of muscle tissues showing a decrease in inflammatory factors and autophagy, downregulation of pyruvate dehydrogenase kinase 4 (*Pdk4*) expression (61.6%) and increased ATP content (25.5%), thereby playing a pivotal role in attenuating muscle degradation in cancer cachexia. Supplementation with inulin diet increased the levels of *Muribaculaceae* and *Muribaculum intestinale* (*p* < 0.05), also alleviating cachexia symptoms in mice.

**Conclusions:**

In cachectic mouse models, *Muribaculaceae* and *Muribaculum intestinale* are reduced and exhibit a significant positive correlation with SCFA butyrate. Inulin or MI supplementation increased these bacteria, ameliorating cachexia. NaB attenuates muscle wasting through coordinated modulation of autophagy suppression, anti‐inflammatory effects and metabolic reprogramming (including PDK4 downregulation and ATP elevation), collectively indicating the existence of a gut–muscle axis in cachexia progression. These findings underscore the potential of microbiota‐targeted interventions in managing cancer cachexia and highlight the intricate interplay between gut microbiota and skeletal muscle health.

## 1 Introduction

Muscle atrophy is one of the major pathological features of cancer cachexia. In recent years, the ‘muscle‐gut axis’ concept has been introduced through academic discussions, suggesting that the gut microbiota could influence muscle mass and function through intermediary metabolites or modulation of intestinal physiology [1]. Research has revealed abnormalities in the gut microbiota composition and metabolic processes in mice with C26‐induced cachexia. Specifically, there is an increased prevalence of *Firmicutes* and a decreased prevalence of *Bacteroidetes*, as well as abnormalities in bile acid metabolism within the intestines [2]. A clinical study on faecal samples from cachectic cancer patients revealed that there are differences in gut microbiota abundance among cachectic cancer patients, and the levels of SCFAs tend to decrease, with acetic acid showing a significant reduction [3]. Although more studies have shown preliminary results of the gut microbiota and metabolites in cancer cachexia [4, 5], the specific mechanisms involved remain unclear.

*Muribaculaceae* (formerly designated *S24–7)*, a family of beneficial bacteria belonging to the order *Bacteroidales*, is commonly found in the intestines of mice [6, 7]. Over 600 bacterial species across 10 genera, including *Muribaculum intestinale* have been identified [8], with limited research on *Muribaculum intestinale*. Emerging evidence underscores the intriguing association between *Muribaculaceae* and longevity [9]. Studies show a significant decrease in *Muribaculaceae* in sarcopenia patients, inversely related to blood lipid levels, suggesting its role in regulating cellular membrane fatty acid composition [10].

The dynamic interplay between intestinal probiotics and prebiotic substrates affects probiotic colonization and proliferation, with dietary fibre being a pivotal prebiotic influencing probiotic growth [11, 12]. As an indigestible carbohydrate, it undergoes fermentation by the gut microbiota, profoundly influencing the microbiome's composition and functionality [13]. Its consumption provides various health benefits, reducing the risk of cardiovascular diseases, hypertension, obesity, Type 2 diabetes and other maladies [14]. Inulin, primarily sourced from inulin‐rich roots, is a soluble fibre garnering significant attention, and numerous investigations have indicated its correlation with the abundance of *Muribaculaceae* [15]. In a leukaemia‐induced cachexia mouse model, inulin‐type fructans with *Lactobacillus reuteri* markedly improved the gut microbiota composition, bolstered intestinal barrier integrity and immune competence and ameliorated cachexia symptoms [16].

SCFAs, resulting from dietary fibre fermentation in the gut, are short‐chain fatty acids with carbon chains of 1–6 atoms, with total colonic concentrations of 50–150 mM [17]. The three most abundant SCFAs are acetic acid, propionic acid and butyric acid [18]. Their production is influenced by gut microbiota and dietary fibre intake. SCFAs contribute to protein synthesis, cellular metabolism, immune modulation, intestinal integrity, lipid metabolism and anti‐inflammatory effects [19]. Some studies suggest SCFAs intake can improve muscle atrophy, though the specific mechanism is unclear [20, 21]. Butyric acid, a four‐carbon metabolic product of gut bacteria, is the most potent anti‐inflammatory SCFA. It reduces TNF‐*α*, inflammation and boosts survival in diseased mice [21].

In this study, we explored the relationships among the gut microbiota, intestinal metabolites and cachexia, focusing on *Muribaculaceae*, *Muribaculum intestinale* and SCFAs in cancer cachexia‐associated muscle atrophy. We investigated the potential therapeutic effects of MI, butyrate and inulin supplementation. Our findings may complement existing research on the gut–muscle axis in cancer cachexia.

## 2 Methods

### 2.1 Cell Culture

C2C12 cells were cultured in DMEM with 10% FBS and 1% P/S until confluent and then differentiated into myotubes in DMEM with 2% horse serum for 5–7 days. Colon‐26 carcinoma (C26) cells were cultured in RPMI 1640 medium with 10% FBS and 1% P/S. Lewis lung carcinoma (LLC) cells were cultured in DMEM with the same supplements. After 48 h, C26 and LLC supernatants were collected, filtered and co‐cultured with myotubes. Sodium butyrate (1 mM) was added 24 h before RNA and protein extraction. All cells were maintained at 37°C with 5% CO_2_.

### 2.2 Animal Models and Experiment

Specific pathogen‐free (SPF) male BALB/c and C57BL/6J mice, aged 6–8 weeks, were obtained from Nanjing Junke Biotechnology Co., Ltd. and housed under a 12h light/dark cycle at 22°C–24°C and 40%–60% humidity with free access to food and water. Cancer cachexia was induced by subcutaneous injection of 5 × 10^5^ C26 cells into BALB/c mice and 5 × 10^5^ LLC cells into C57BL/6J mice, respectively [22]. The inulin diet and control diet were prepared by Nanjing Junke Biotechnology Co., Ltd., based on published literature [23]. Details of different animal experiments are described in the Supporting Information.

### 2.3 Quantitative Real‐Time PCR (qPCR)

Total RNA was extracted from frozen muscle tissue or cells using a total RNA extraction reagent. After determining the RNA concentration using a NanoDrop, 1 μg was reverse transcribed into cDNA. SYBR Green qPCR mix was used with *β* *‐actin* as an internal reference; primer sequences for all target genes are in Table S2.

### 2.4 16S rRNA Gene Amplicon Sequencing and Bioinformatics

Collected mouse faeces were immediately snap‐frozen in liquid nitrogen and preserved at −80°C. Sequencing and analysis were performed by Wuhan MetWare Metabolic Biotechnology Co., Ltd. [24]. Details are described in the Supporting Information.

### 2.5 Short‐Chain Fatty Acid Assessment

Mouse faeces were collected and stored as described above. SCFAs content was determined by Wuhan MetWare Metabolic Biotechnology Co., Ltd. using the Agilent 7890B‐7000D GC–MS/MS platform. Details are described in the Supporting Information.

### 2.6 RNA Sequencing and Bioinformatics

The total RNA extracted from mouse skeletal muscle was subjected to RNA sequencing on the Illumina Novaseq 6000 platform by LC Bio Technology Co., Ltd. Details of the bioinformatics are described in the Supporting Information.

Other methods such as *Muribaculum intestinale* culture, haematoxylin and eosin (H&E) staining, immunofluorescence staining, protein extraction, Western blotting, ATP content measurement and untargeted metabolomics of muscle are described in the Supporting Information.

### 2.7 Statistical Analysis

The data are presented as the mean ± SEM. Two‐tailed Student's *t* tests were used for comparisons between two groups. One‐way ANOVA tests were performed to compare multiple groups. The Spearman's correlation analysis was used to compute the association between differentially abundant microbial taxa and differential SCFAs. The analyses were performed using GraphPad Prism 8.0.1 and R 4.3.0. Values of *p* < 0.05 were considered to be statistically significant and were presented as *p < 0.05, **p < 0.01, ***p < 0.001; ns indicates non‐significant.

## 3 Results

### 3.1 Alterations in Gut Microbiota Composition in Cachexia Model Mice

**FIGURE 1:**
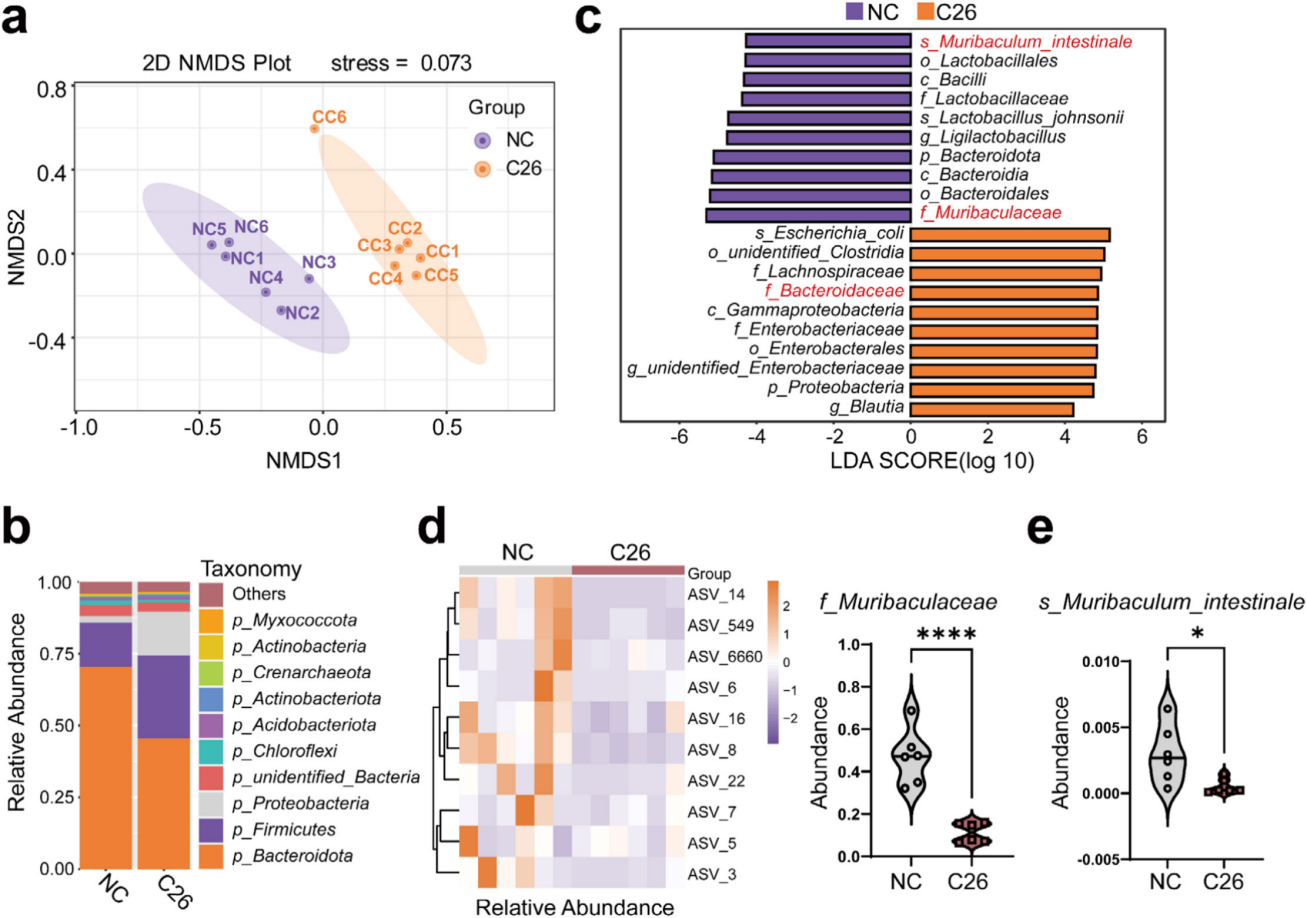
Abnormal gut microbiota distribution and reduced *Muribaculaceae* abundance in cachexia mouse model. (a) Nonmetric multidimensional scaling (NMDS) plot illustrating the similarities in gut microbiota between control and cachectic mice. The purple and orange shadows represent clustering within the groups (*n* = 6). (b) Stacked bar‐plot showing the top 10 most abundant bacterial at phylum level between control and cachectic mice. (c) The differences in gut microbiota abundance between the control and cachectic groups using linear discriminant analysis effect size (LEfSe) analysis. (d) Heatmap analysis reveals the distribution of various Amplicon Sequence Variants (ASVs) within the microbiome *f_Muribaculaceae*. The right panel represents the abundance of *f_Muribaculaceae*. (e) The abundance of *Muribaculum intestinale* within the groups (*n* = 6). Two‐tailed unpaired Student's *t* tests was used. Statistical significance: *p < 0.05; ****p < 0.0001.

To elucidate the impact of gut microbiota on cancer cachexia, faecal samples from two murine models exhibiting cachexia were subjected to comprehensive 16S rRNA gene amplicon sequencing analysis. In the C26 cachexia model, alpha diversity, including Chao1, Shannon, ACE and Simpson indexes, showed no significant variation between control and cachexia groups, implying stability in microbial richness and evenness in cachexia (Figure S1a). In contrast, beta diversity analysis using nonmetric multidimensional scaling (NMDS) based on the amplicon sequence variant (ASV) revealed distinct microbial community clustering between control and cachexia mice (Figure 1a), indicating substantial structural changes. Further, a detailed taxonomic composition analysis revealed significant shifts at the phylum level. There was an increase in the relative abundances of *p_Firmicutes* and *p_Proteobacteria* and a decrease in *p_Bacteroidota* in the cachexia model (Figure 1b). LEfSe analysis revealed differentially abundant microbial taxa in cachexia mice, with enrichment of *s_Escherichia_coli*, *o_unidentified_Clostridia*, *f_Lachnospiraceae*, *f_Bacteroidaceae* and *c_Gammaproteobacteria*, and reduction of *f_Muribaculaceae*, *o_Bacteroidales*, *c_Bacteroidia* and *s_Muribaculum intestinale* (Figure 1c). Tax4Fun2‐based functional predictions from ASV data indicated significant enrichment in cellular processes, environmental information processing and various other biological and disease pathways (Figure S1b). The 16S rRNA gene amplicon sequencing revealed pronounced decreases in *Muribaculaceae* and *Muribaculum intestinale* in the cachexia group compared to *p_Bacteroidota* and *o_Bacteroidales* (Figure S1c–f). Specifically, *Muribaculaceae* and *Muribaculum intestinale* abundances dropped by 76.0% (Figure S1c) and 82.0% (Figure S1d), respectively, while *p_Bacteroidota* and *o_Bacteroidales* showed smaller reductions of approximately 35.4% (Figure S1e) and 36.3% (Figure S1f), respectively. Diverse *Muribaculaceae* species display substantial V4 16S rRNA gene sequence variation (Figure 1d), with *Muribaculum intestinale* being one of the few identified (Figure 1e). Its reduced abundance in cancer cachexia implicates a significant role for *Muribaculaceae*, particularly *Muribaculum intestinale*, in cachexia pathogenesis.

In the LLC cachexia model, alpha diversity indices showed increased microbial diversity (Figure S1g), and beta diversity highlighted intergroup differences (Figure S1h). Phylum‐level analysis revealed increased *Firmicutes* and a decrease in *Bacteroidota* (Figure S1i). LEfSe analysis indicated enrichment of *g_Bacteroides*, *s_Bacteroides_sartorii* and *f_Bacteroidaceae* and a reduction in *f_Muribaculaceae*, *s_Faecalibaculum_rodentium*, *s_Muribaculum intestinale* and *s_Bifidobacterium_pseudolongum* in LLC cachexia mice (Figure S1j). Functional annotation clustering confirmed gene enrichment in key pathways, including those for cellular processes and environmental information processing (Figure S1k). Our results reveal a significant alteration in gut microbiota in mouse models of cachexia, characterized primarily by a substantial decrease in *Muribaculaceae* and *Muribaculum intestinale*.

### 3.2 Muribaculum Intestinale Supplementation Mitigates Cancer Cachexia

**FIGURE 2:**
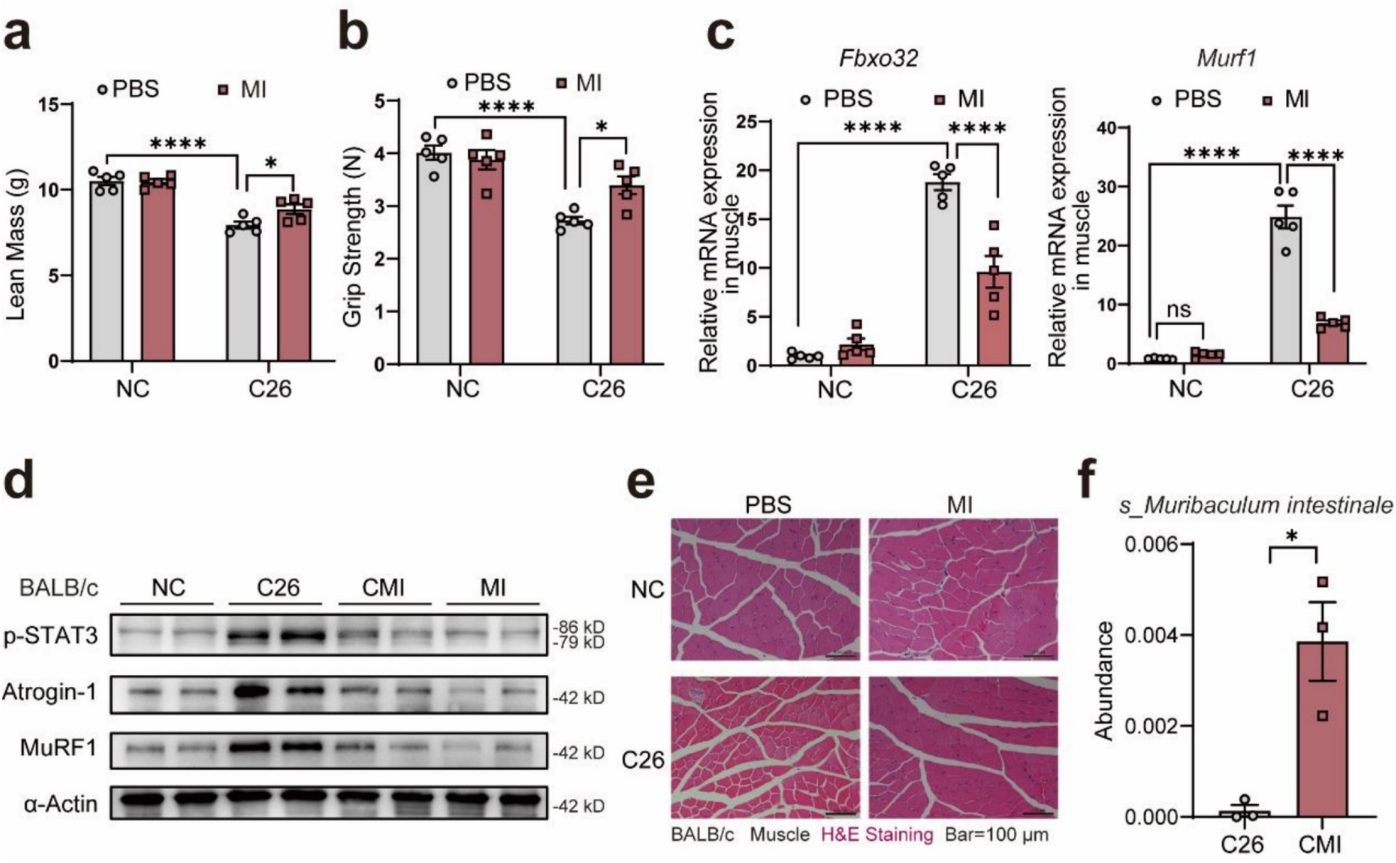
Supplementation with *Muribaculum intestinale* mitigates C26 cancer cachexia. (a) Weight of lean mass showing the changes in *Muribaculum intestinale* (MI) supplemented mice (*n* = 5). (b) Grip strength of mice in each group before the end of the experiment (*n* = 5). (c) Relative mRNA expression of *Fbxo32* and *Murf1* in muscle from each group (*n* = 5). (d) The protein expression of Atrogin‐1, MuRF1 and p‐STAT3 in the muscle of MI supplemented mice. (e) H&E staining of the quadriceps muscle shows MI supplementation's effect on muscle. The scale bar represents 100 μm. (f) The abundance of the MI after supplementation (*n* = 5). The data are presented as the mean ± SEM. One‐way ANOVA and two‐tailed unpaired Student's *t* tests were used. Statistical significance: *p < 0.05, ****p < 0.0001.

We evaluated the impact of *Muribaculum intestinale*, a *Muribaculaceae* family member, on a cachectic mouse model. Anaerobically cultured to a concentration of 10^9^ CFU/mL, the strain was administered at 100 μL via gavage. Treatment with MI significantly improved cachexia, reducing weight loss (Figure S2a), preserving lean mass (Figure 2a), hindlimb muscles (Figure S2b) and quadriceps weight (Figure S2c) and enhancing grip strength (Figure 2b), with no effect on tumor size (Figure S2d).

MI treatment downregulated mRNA (Figure 2c) and protein (Figures 2d and S2e) levels of muscle atrophy markers Atrogin‐1 (*Fbxo32* in mRNA) and MuRF1 (*Murf1* in mRNA) in cachectic mice. This was paralleled by a reduction in p‐STAT3 (Figures 2d and S2e), suggesting an anti‐inflammatory effect and amelioration of muscle atrophy. H&E staining indicated muscle fibre preservation, with an increased cross‐sectional area (CSA) following treatment (Figures 2e and S2f). 16S rRNA gene amplicon sequencing confirmed elevated *Muribaculum intestinale* levels post‐administration (Figure 2f).

In the LLC cachexia model, MI treatment led to significant preservation of lean mass (Figure S2g) and hindlimb muscles (Figure S2h), and enhanced grip strength (Figure S2i), with no change in tumour weight (Figure S2j). H&E staining confirmed muscle fibre protection, as evidenced by increased CSA (Figure S2k). qPCR and Western blotting results mirrored those from C26 mice, showing reduced expression of muscle atrophy markers (Figure S2l, S2m). The 16S rRNA gene amplicon sequencing revealed increased faecal *Muribaculum intestinale* levels post‐treatment in LLC mice (Figure S2n). These findings suggest that *Muribaculum intestinale*, as a potential probiotic, can positively affect cachectic mouse models.

### 3.3 Decrease in SCFAs Associated With Reduced Muribaculaceae and Muribaculum Intestinale Abundance in Cachexia Mice

**FIGURE 3:**
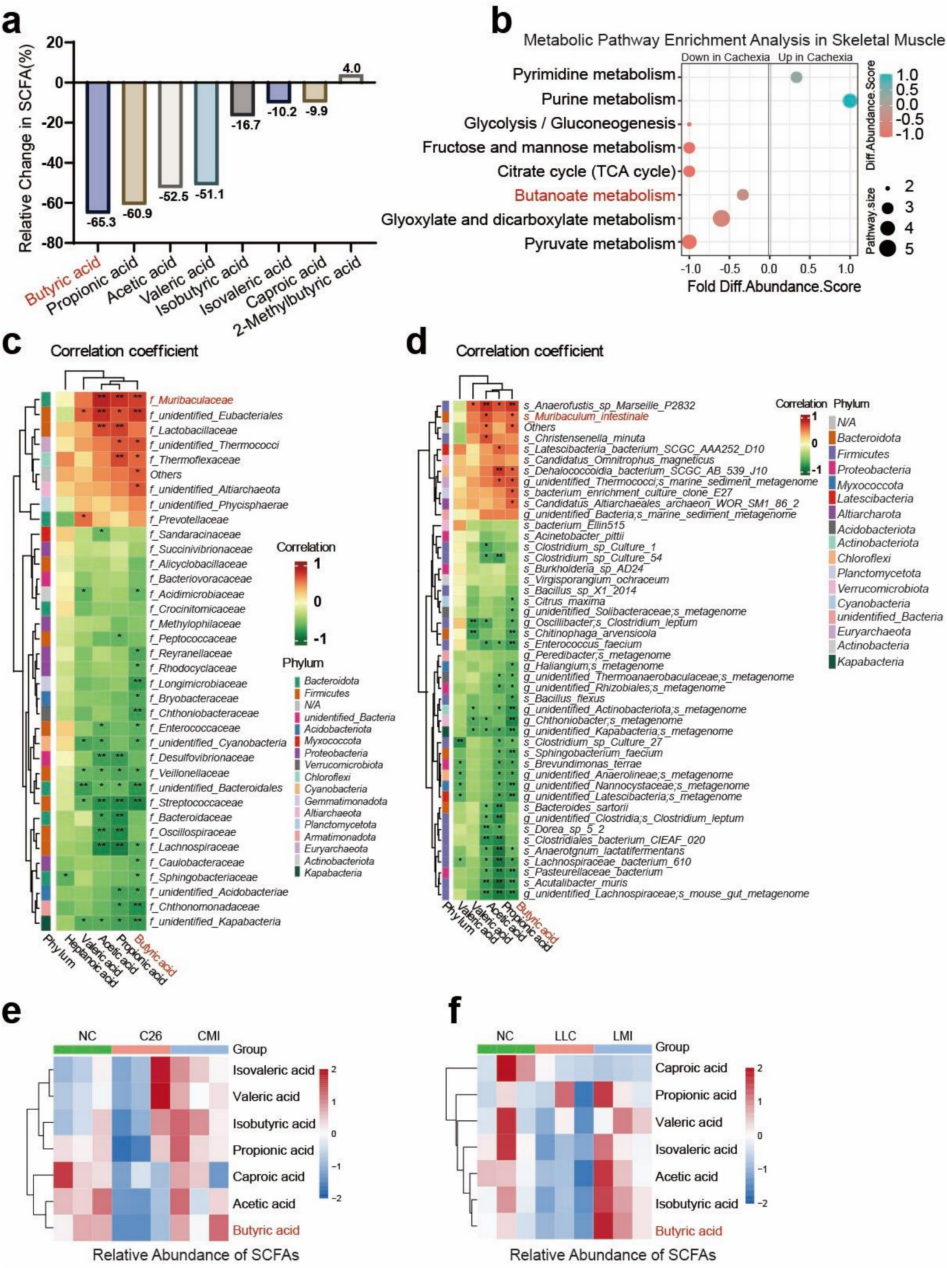
Decreased short‐chain fatty acids (SCFAs) levels in the faecal samples of cachexia mice correlate positively with *Muribaculaceae* abundance. (a) Percentage changes of SCFAs in the cachexia group compared to the control group. (b) Bubble chart showing pathway enrichment of altered metabolites in the muscle of C26 cachexia mice. (c) The correlation heatmap at the family level. (d) The correlation heatmap at the genus and species level (*n* = 6). (e,f) The heatmaps showing the SCFAs changes with MI supplementation in C26 cachectic mice (e) and LLC cachectic mice (f) (*n* = 3). The data are presented as the mean ± SEM. Spearman's correlation analysis was used. Statistical significance: *p < 0.05; **p < 0.01.

To investigate the potential consequences of the observed gut dysbiosis, we performed SCFAs analysis on faecal samples from C26 cachexia mice and healthy controls, and observed a significant reduction in SCFAs in the cachectic group: acetic acid (AA) by 52.5%, propionic acid (PA) by 60.9%, and valeric acid (VA) by 51.1%. Notably, butyric acid (BA) showed the most substantial decrease, at 65.3%. Levels of isovaleric acid (IVA), isobutyric acid (IBA), caproic acid (CA) and 2‐methylbutyric acid (2‐BA) remained unchanged (Figures 3a and S3a). Muscle tissue metabolomics revealed a clear separation between the NC and C26 cachexia groups in principal component analysis (PCA) (Figure S3b, S3c) and distinct clustering in metabolite heatmaps (Figure S3d,S3e), accompanied by downregulation in butanoate metabolism pathways (Figure 3b), suggesting a systemic impact of altered SCFA profiles. This decrease in butanoate metabolism suggests its critical role in cachexia, emphasizing its importance in the condition's pathophysiology.

To determine the correlation between microbial taxa shifts and metabolite profiles, we performed a Spearman correlation hierarchical clustering analysis. This analysis disclosed significant associations between gut microbiota at various taxonomic levels and SCFAs. At the phylum level, significant reductions in *Bacteroidota*, *Euryarchaeota*, *Altiarchaeota* and *Crenarchaeota* were positively correlated with decreases in specific SCFAs. In contrast, *Myxococcota* and *Firmicutes* showed negative correlations with SCFA levels (Figure S3f). At the class and order levels, we highlighted taxa such as *Thermoplasmata*, *Limnochordia* and *Thermococci*, which exhibited decreased abundance and positive correlations with certain SCFAs. Conversely, the abundance of classes such as *Clostridia* and *Negativicutes* increased, showing negative correlations with SCFA levels (Figure S3g). Similarly, at the order level, *Eubacteriales* and *Bacteroidales* significantly decreased and positively correlated with several SCFAs, while unidentified taxa within the *unidentified Clostridia* and *Kapabacteria* orders were negatively correlated with these metabolites (Figure S3h). At the family level, a significant decrease in *Muribaculaceae*, along with reduced abundances of *unidentified Eubacteriales* and *Lactobacillaceae*, was positively correlated with several differential SCFAs, particularly butyric acid (Figure 3c). Increases in families such as *Chthonomonadaceae*, *Sphingobacteriaceae*, *Lachnospiraceae* and *Oscillospiraceae* were associated with negative correlations to SCFA levels (Figure 3c). At the genus and species levels, decreases in taxa such as *Anaerofustis* sp. *Marseille‐P2832*, *Muribaculum intestinale* and *Christensenella minuta* were significantly correlated with declines in several differential SCFAs. In contrast, increases in *unidentified Lachnospiraceae*, *mouse gut metagenome*, *Acutalibacter muris* and *Pasteurellaceae bacterium* were negatively correlated with these metabolites (Figure 3d). These findings indicate a profound disruption in both the composition and functional capacity of the gut microbiota in cachexia, characterized by a marked decline in SCFA levels, particularly BA. The strong correlation between *Muribaculaceae* and BA levels, as well as between *Muribaculum intestinale* and BA levels highlights the significance of these bacteria in SCFA production. Interestingly, MI supplementation in two cachectic mouse models led to significant changes in faecal SCFA profiles, particularly an increase in butyrate levels (Figure 3e, 3f). These results suggest that *Muribaculum intestinale* may be pivotal in modulating the host butyrate concentrations.

### 3.4 Sodium Butyrate Supplementation Mitigates Cachexia Symptoms and Suppresses Muscle Degradation

**FIGURE 4:**
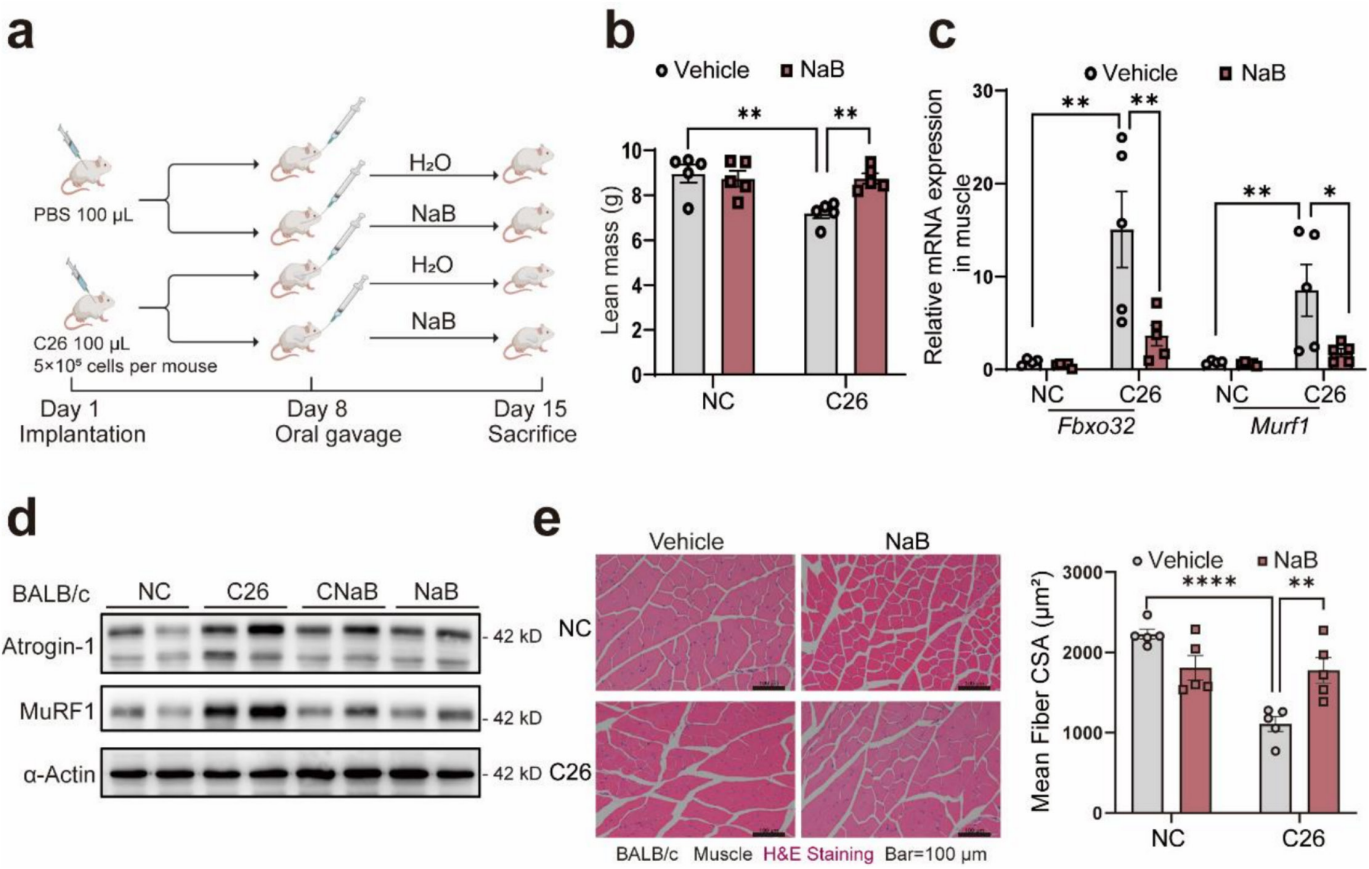
Sodium butyrate supplementation alleviates weight loss and suppresses muscle degradation in cachexia mice. (a) Schematic of sodium butyrate (NaB) supplementation in cachexia mice. (b) Weights of lean mass and hind limb muscle changes in NaB‐supplemented mice (*n* = 5). (c) Relative mRNA expression of *Murf1* and *Fbxo32* in muscle with NaB supplementation (*n* = 5). (d) The protein expression of Atrogin‐1, MuRF1 in muscle of NaB‐supplemented mice, with statistics analysis is shown on the right panel. (e) H&E staining of the quadriceps muscle shows the effect of NaB supplementation on muscle, with statistical analysis shown on the right. The scale bar represents 100 μm. The data are presented as the average ± SEM. One‐way ANOVA was used. Statistical significance: *p < 0.05; **p < 0.01; ****p < 0.0001.

Given the depletion of SCFAs, especially BA, in cachexia, we explored the therapeutic effects of sodium butyrate (NaB) supplementation in the C26 cachexia model (Figure 4a). Treatment resulted in amelioration of the cachexia phenotype, including reduced body weight loss (Figure S4a). Although on Day 13, there was an upward trend in body weight in the CNaB group of mice, no significant difference was observed compared to Day 8 (Figure S4b) or the NaB control group on Day 13 (Figure S4c). Lean body mass and hindlimb muscle were preserved (Figures 4b and S4d), despite a reduction in tumour weight (Figure S4e). Molecular analyses demonstrated reduced mRNA levels of atrophy markers *Fbxo32* and *Murf1* in NaB‐treated cachectic mice (Figure 4c). Western blotting confirmed decreased protein expression of the cachexia‐specific markers Atrogin‐1 and MuRF1 (Figures 4d and S4f), suggesting effective alleviation of muscle atrophy. H&E staining showed increased CSA of muscle fibres and adipose tissues in NaB‐treated cachectic mice, indicating a reduction in tissue degradation (Figures 4e and S4g). In the LLC cachexia mouse model, NaB supplementation increased the muscle fibre CSA (Figure S4h) and suppressed muscle atrophy markers (Figure S4i, S4j). Unlike the C26 model, tumour weight remained unchanged in the LLC model with NaB treatment (Figure S4k, S4l), suggesting that its effects on tumorigenesis may vary between cachexia models.

**FIGURE 5:**
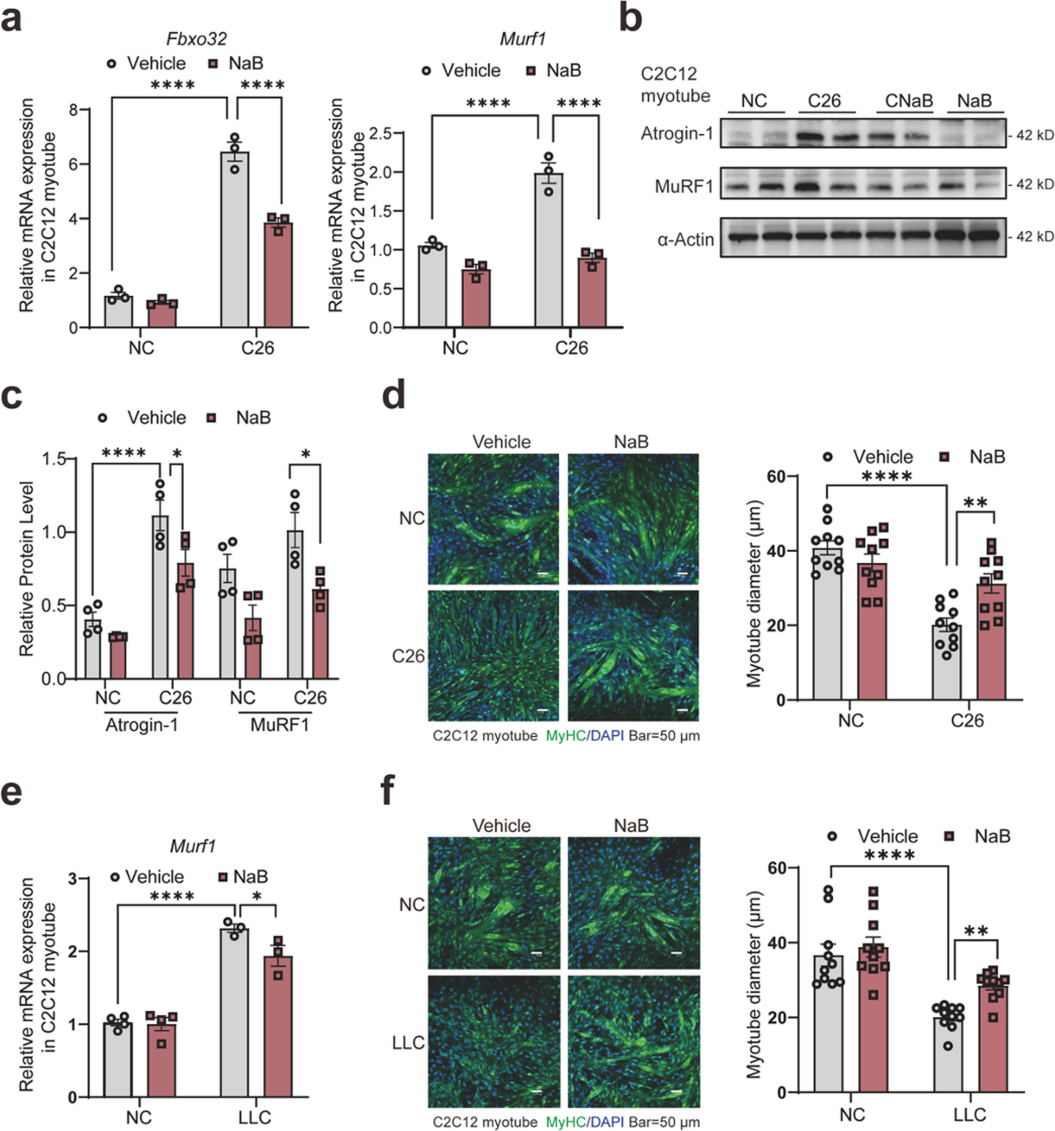
Sodium butyrate can improve cachexia‐induced myotube atrophy *in vitro* *.* (a) Relative mRNA expression of *Fbxo32* and *Murf1* in C2C12 myotubes with NaB treatment (*n* = 3). (b) Protein expression of Atrogin‐1, MuRF1 in C2C12 myotubes treated with NaB. (c) The statistics analysis of Figure 5b (*n* = 4). (d) Immunofluorescence staining of MyHC in the C26 cachexia cell model with NaB treatment, and quantitative analysis of myotube diameter on the right panel, the scale bar represents 50 μm. (e) Relative mRNA expression of *Murf1* in myotubes in the LLC cachexia model treated with NaB (*n* **=** 3). (f) Immunofluorescence staining of MyHC in the LLC cachexia cell model with NaB treatment, and quantitative analysis of myotube diameter on the right panel, the scale bar represents 50 μm. The data are presented as the mean ± SEM. One‐way ANOVA was used. Statistical significance: *p < 0.05; **p < 0.01; ****p < 0.0001.

We also validated this in the C2C12 myotube. NaB treatment decreased atrophy markers (Figure 5a–5c) and protected myotubes from C26 conditional medium, as shown by immunofluorescence (Figure 5d). The protective effect was consistent when myotubes were treated with LLC conditional medium, showing a notable decrease in *Murf1* expression (Figure 5e) and protection against myotube shrinkage (Figure 5f). The findings show that NaB not only reduces cachexia‐related muscle and fat loss *in vivo*, but also directly affects muscle atrophy pathways.

### 3.5 Sodium Butyrate Induces Transcriptomic Shifts That Counteract Cachexia‐Associated Muscle Atrophy Pathways

**FIGURE 6:**
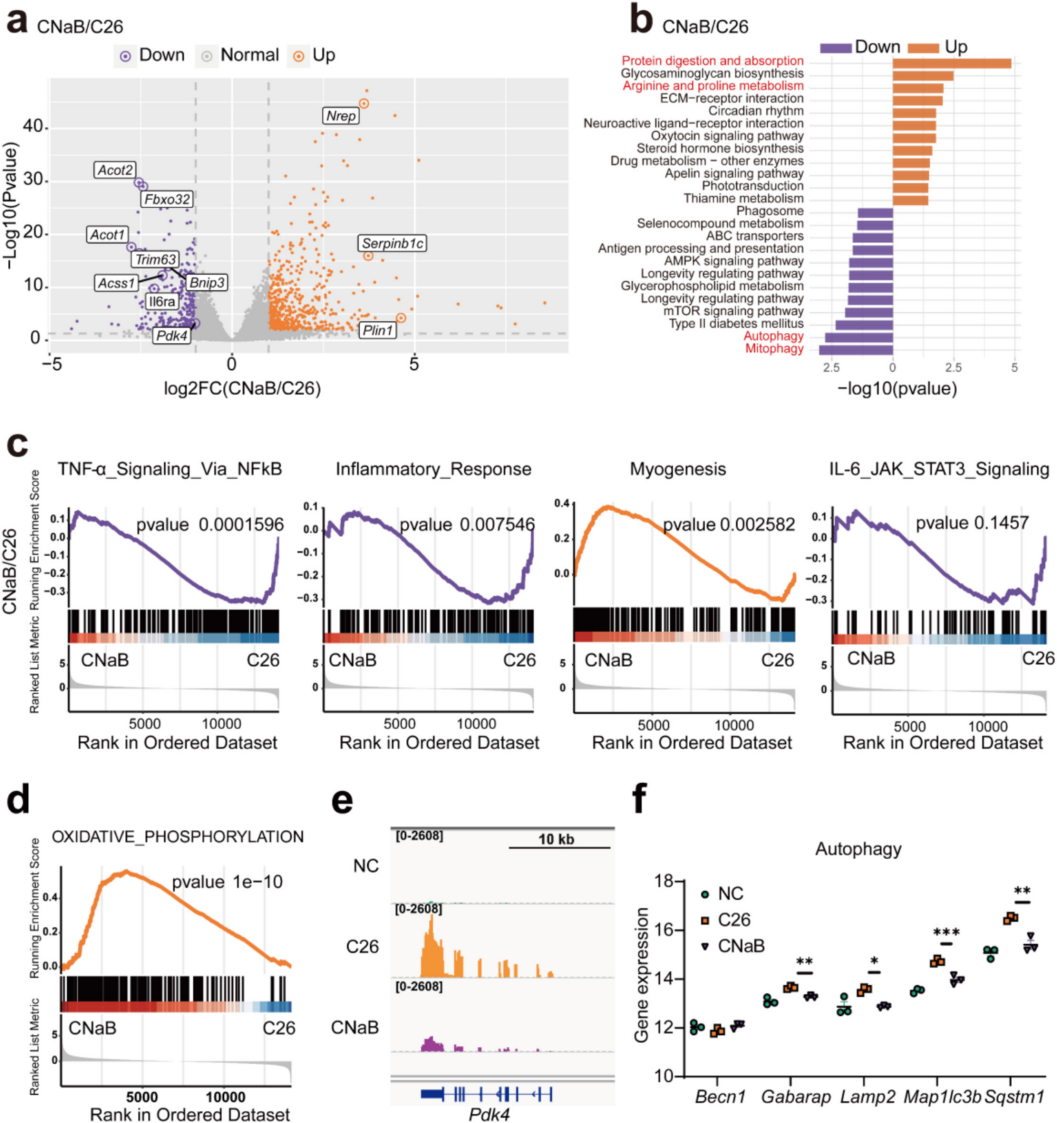
Muscle RNA‐Seq demonstrated that sodium butyrate treatment can affect the expression levels of multiple genes in cachexia skeletal muscle samples. (a) Volcano plot showing the significantly changed genes with NaB treatment; the orange is upregulated genes in the CNaB group and the purple is the downregulated genes in the same group. (b) KEGG pathway analysis showing the most featured pathway enrichment in the CNaB and C26 groups. (c) Gene set enrichment analysis (GSEA) of TNF‐*α* signalling, inflammatory response, IL‐6‐JAK‐STAT3 signalling and myogenesis in the CNaB and C26 groups. (d) GSEA of oxidative phosphorylation. (e) The representative of *Pdk4* mRNA expression in three groups. (f) Analysis of autophagy‐related genes in mouse muscles in three groups (−rpkm) (*n* = 3). The data are presented as the mean ± SEM. One‐way ANOVA was used. Statistical significance: *p < 0.05; **p < 0.01; ***p < 0.001.

To clarify NaB's role in alleviating cachexia‐associated muscle atrophy, we performed RNA‐Seq on quadriceps muscle. PCA showed the NaB‐treated group's gene expression was closer to normal controls than the untreated group (Figure S5a). The heatmap revealed significant gene expression differences among groups, indicating substantial transcriptomic changes from NaB (Figure S5b). NaB downregulated key atrophy‐related genes like *Trim63*, *Fbxo32*, *Acot1*, *Acot2*, *Pdk4* and *Il6ra* (Figure 6a), which are typically upregulated in cachectic muscle (Figure S5c), suggesting a direct impact on muscle preservation. Kyoto Encyclopedia of Genes and Genomes (KEGG) pathway analysis showed that in the cachexia group, pathways such as mitophagy and FoxO signalling were upregulated, while glycolysis and glycine, serine and threonine metabolism were downregulated (Figure S5d). Remarkably, NaB reversed these trends by reducing activity in autophagy‐related pathways and enhancing pathways associated with arginine and proline metabolism, as well as glycosaminoglycan biosynthesis (Figure 6b). Gene ontology (GO) enrichment analysis revealed significant alterations in biological processes, including the regulation of nervous system development, axon growth and wound response, underscoring the extensive systemic impact of NaB (Figure S5e). Regarding cellular components and molecular functions, there was notable enrichment in extracellular matrix structures and calcium ion binding activities, among other aspects (Figure S5f,S5g). Gene set enrichment analysis (GSEA) showed that in cachexia, TNF‐*α*, inflammatory and JAK‐STAT3 pathways were upregulated, and myogenesis pathways were downregulated (Figure S5h). NaB significantly suppressed TNF‐*α* and inflammatory pathways, mildly affected the JAK‐STAT3 pathway, and enhanced muscle generation pathways (Figure 6c). It also activated oxidative phosphorylation (Figure 6d). These findings were associated with coordinated transcriptional changes, including the downregulation of *Pdk4* (Figure 6e) and autophagy‐related genes (Figures 6f and S5i), collectively highlighting NaB's multifaceted modulation of inflammatory signalling and energy metabolic rewiring in cachectic muscle.

### 3.6 Sodium Butyrate Suppresses STAT3 Signalling and Autophagy, Supporting Transcriptomic Findings

**FIGURE 7:**
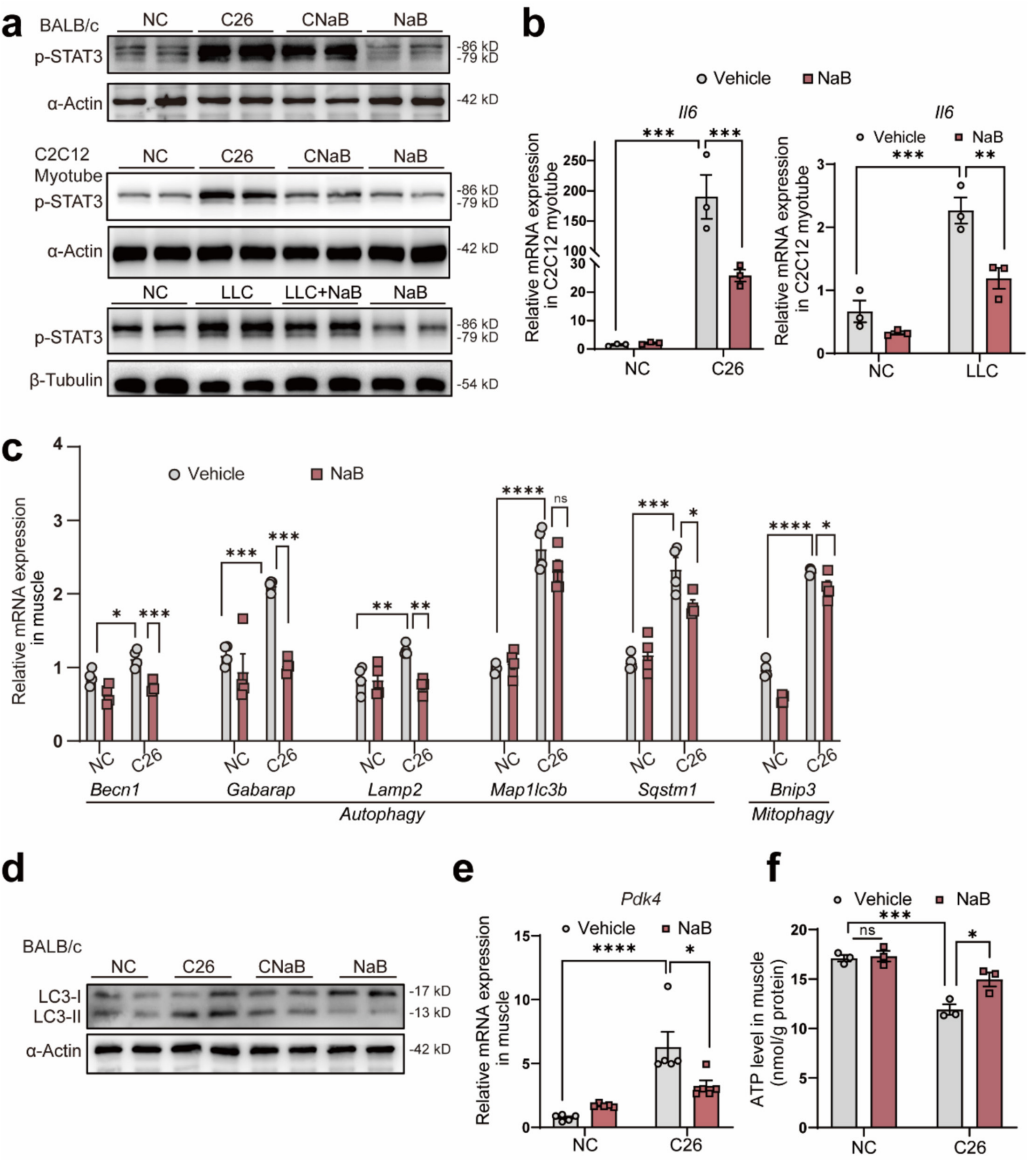
Experimental validation of the transcriptomic data results. (a) Protein expression of pSTAT3 in different cachexia models treated with NaB. (b) Relative mRNA expression of *Il6* in C2C12 myotubes with NaB treatment (*n* = 3). (c) Relative mRNA expression of autophagy and mitophagy‐related genes in each group (*n* = 4). (d) Protein expression of LC3 in C2C12 myotubes with NaB treatment. (e) Relative mRNA expression of *Pdk4* expression in muscle with NaB treatment (*n* = 5). (f) ATP quantification in C26 cachectic mice with NaB treatment (*n* = 3). The data are represented as the mean± SEM. One‐way ANOVA was used. Statistical significance: ns means no significance, *p < 0.05; **p < 0.01; ***p < 0.001; ****p < 0.0001.

To validate key findings, we investigated NaB's effects on inflammation and autophagy signalling, both implicated in cachexia‐induced muscle wasting. Western blotting results revealed a significant reduction in p‐STAT3 levels in cachectic mice's skeletal muscles and myotubes treated with NaB (Figure 7a). qPCR analysis revealed that NaB reduced the elevation of *Il6* mRNA levels in myotubes induced by C26 and LLC conditional medium (Figure 7b). The multiplex assay kit assessed plasma inflammatory cytokines in both cachexia mouse models. In the C26 model, NaB significantly reduced TNF‐*α*, IL‐6, IL‐1*β* and MCP‐1 (Figure S6a–S6d). In the LLC model, IL‐6 and MCP‐1 decreased, but TNF‐*α* and IL‐1*β* showed no significant change (Figure S6e–S6h). These findings indicate that NaB can mitigate cachexia‐associated systemic inflammation.

Further qPCR analysis showed that NaB treatment significantly downregulated mRNA levels of autophagy and mitophagy‐specific genes, indicating reduced catabolic activity (Figure 7c). Western blotting confirmed this finding, showing a decreased LC3 II/I ratio and reduced autophagy activity in muscle tissues (Figure 7d). The qPCR results showed a significant decrease in *Pdk4*, which is involved in inhibiting oxidative phosphorylation (Figures 7e and S6i). Interestingly, in the previous experiment, MI supplementation led to increased butyrate levels (Figure 3e,3f), and qPCR analysis of muscle samples from cachectic mice supplemented with MI showed significant suppression of *Pdk4* expression (Figure S6j), similar to direct NaB supplementation. Increased ATP content confirmed the enhancement of oxidative phosphorylation in muscles following NaB treatment (Figure 7f).

### 3.7 Inulin Supplementation Rebalances Gut Microbiota and Mitigates Cancer Cachexia

**FIGURE 8:**
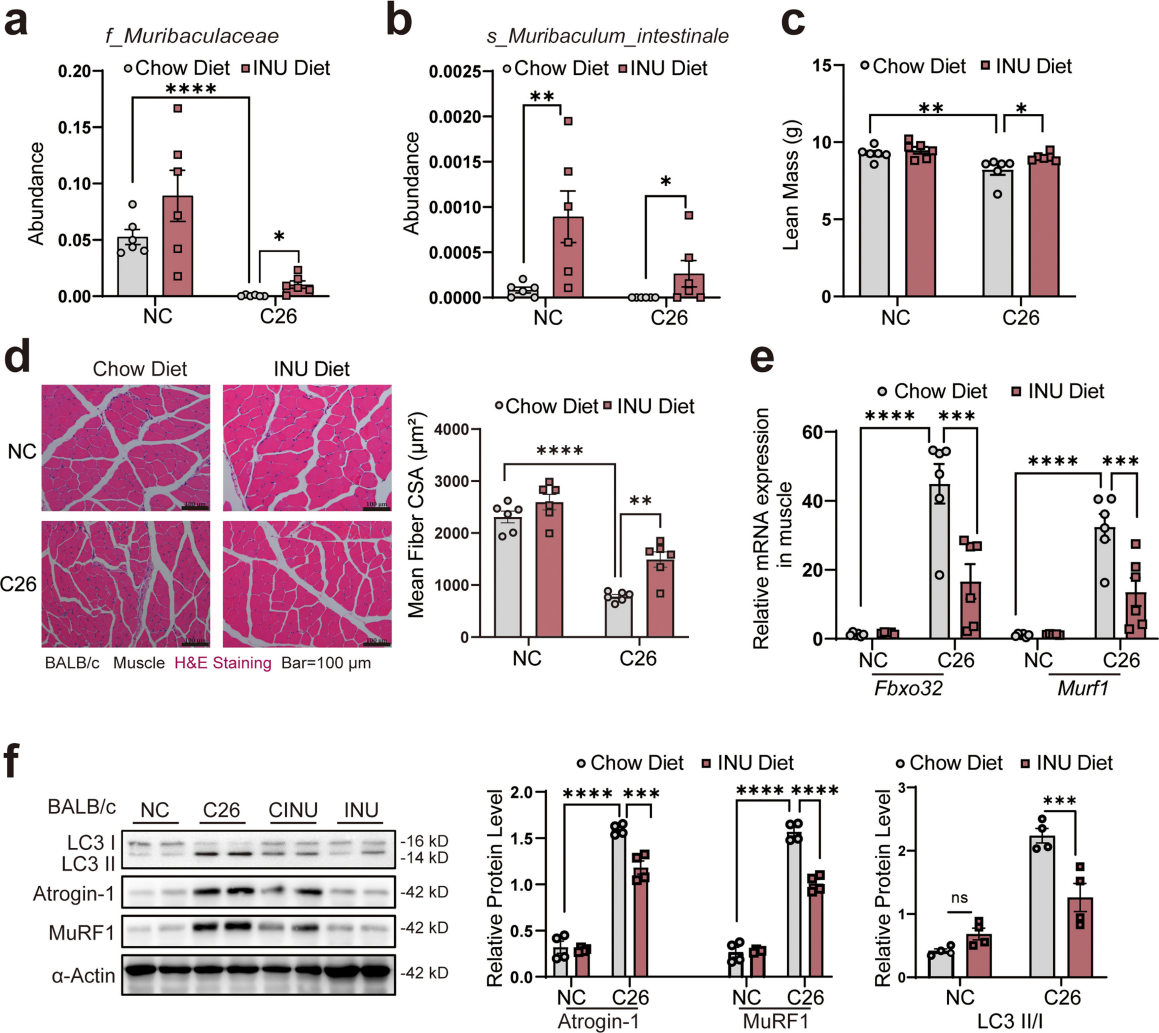
Supplementation with inulin restored intestinal microbiota stability, enhanced *Muribaculaceae* abundance, and ameliorated cancer cachexia. (a,b) The abundance of *f_Muribaculaceae* and *s_Muribaculum intestinale* in each group (*n* = 6). (c) Weight of lean mass showing the changes in inulin‐supplemented mice (*n* = 6). (d) H&E staining of quadriceps muscle showing the effect of inulin supplementation on muscle, the statistics analysis is shown on the right panel. The scale bar represents 100 μm. (e) Relative mRNA expression of *Murf1* and *Fbxo32* in muscle from each group (*n* = 6). (f) The corresponding protein expression and the statistics analysis of Atrogin‐1, MuRF1 and LC3 in the muscle of inulin‐supplemented mice. The data are presented as the mean ± SEM. One‐way ANOVA was used. Statistical significance: ns means no significance, *p < 0.05; **p < 0.01; ***p < 0.001; ****p < 0.0001.

Regarding clinical application potential, inulin might have an advantage in safety, transport and patient agreement. Thus, we employed an inulin fructan‐enriched prebiotic diet that is known to increase the abundance of *Muribaculaceae* [15, 23]. To expand the protective effect against cancer cachexia, we administered an inulin fructan‐enriched diet to the cachexia model mice (Figure S7a). 16S rRNA gene amplicon sequencing revealed that inulin supplementation improved cachexia‐associated microbial dysbiosis (Figure S7b). At the phylum level, *Firmicutes* in cachectic mice decreased following inulin intervention, while the proportion of *Bacteroidota* increased (Figure S7c). At the class level, *Bacteroidia* and *Clostridia* showed an upward trend (Figure S7d). At the order level, *Bacteroidales* and *Lachnospirales* also increased (Figure S7e). At the family level, *Muribaculaceae* increased significantly (Figures S7f and 8a). At the species level, *Muribaculum intestinale* increased significantly (Figure 8b). These changes suggest that inulin may improve gut microbiota balance in cachexia, benefiting intestinal health.

The prebiotic intervention notably improved the cachexia phenotype, including reduced body weight loss (Figure S7g), preserving lean mass (Figure 8c) and quadriceps muscles (Figure S7h) and hindlimb muscle (Figure S7l). Importantly, these benefits occurred without altering tumour weight (Figure S7i), adipose tissue mass (Figure S7j, S7l) and heart weight (Figure S7k), possibly indicating muscle‐specific effects. H&E staining showed increased muscle fibre CSA in the quadriceps (Figure 8d), with no significant change in adipose tissue CSA (Figure S7m), indicating an effective counteraction of muscle atrophy by the inulin diet. Then qPCR of muscle RNA showed reduced expression of *Fbxo32* and *Murf1* in the cachexia model mice fed the inulin diet (Figure 8e). Western blotting confirmed decreased protein levels of Atrogin‐1 and MuRF1, and a reduced LC3II/I ratio (Figure 8f). These results indicate that inulin effectively normalized gut microbiota composition, enhanced *Muribaculaceae* and *Muribaculum intestinale* abundance and significantly improved key cachexia markers.

## 4 Discussion

Cachexia is a complex metabolic syndrome with multifaceted organ changes, accompanied by gut microbiota dysbiosis and significant metabolic abnormalities [2, 3]. While research on specific microbes' impact on muscle function in cachexia is limited [5]. Our study reveals a notable reduction in *Muribaculaceae* and *Muribaculum intestinale* in cachexia mouse models. Direct MI supplementation enhances its gut presence and alleviates cachexia symptoms and increases the levels of butyrate in the faeces of mice, linking *Muribaculaceae* and butyrate [9, 25]. NaB also reduced muscle and fat wasting in cachexia, underscoring the importance of gut microbiota and its metabolites. Inulin increases these gut bacteria and ameliorates the cachexia phenotype. These findings emphasize the gut microbiota's importance in cachexia and offer new treatment strategies through microbiota modulation (Figure S8).

In two cancer cachexia mouse models, we observed significant gut microbiota changes, notably reduced *Muribaculaceae* abundance, with *Muribaculum intestinale* as the key species. These findings are supported by studies showing a significant drop in *Muribaculaceae* in sarcopenia patients, underscoring its potential key role in muscle dysfunction diseases [10]. The consistent findings suggest that the *Muribaculaceae* family and *Muribaculum intestinale* could be crucial for muscle health regulation. In cachectic mice, we observed increased *Firmicutes* phylum abundance alongside decreased *Bacteroidetes* phylum levels. While early obesity studies emphasized phylum‐level compositional shifts (e.g., increase in *Firmicutes* and a decrease in *Bacteroidota* [26]), subsequent research revealed contradictory trends [27], and recent critiques highlight the limited biological relevance of such broad taxonomic ratios given phylum‐level functional heterogeneity [28]. Notably, *Muribaculaceae* (*Bacteroidetes* phylum) depletion may exhibit stronger associations with cachectic phenotypes than phylum‐level changes. In db/db mice, reduced *Bacteroidetes* abundance correlated with muscle weakness [29], paralleling our findings and cancer cachexia reports of *Firmicutes* dominance [3]. This emphasizes the need to further explore the gut microbiota's role in cachexia and its modulation to improve cachexia.

Butyrate, produced by gut microbiota, supplies energy to intestinal epithelial cells and enhances intestinal barrier function by lowering intracellular oxygen levels [30]. It also has systemic anti‐inflammatory effects when absorbed after oral administration [31, 32]. Pötgens et al. found decreased acetate and butyrate in the C26 model [33]. Transcriptome data showed no significant IL‐6‐STAT3 suppression by NaB, but experiments confirmed its inhibition in muscles. Interestingly, in the muscles of mice treated with MI and NaB, the mRNA levels of *Pdk4* were significantly decreased. Although the critical role of PDK4 in muscle atrophy has been supported by multiple independent studies [34, 35], where elevated PDK4 expression positively correlates with muscle wasting and its inhibition ameliorates atrophy, this study has not yet directly validated the causal relationship through genetic knockout/overexpression experiments. These findings suggest that the improvement of cachexia‐induced muscle atrophy by MI and butyrate may potentially involve PDK4 suppression, but this mechanistic link requires further experimental confirmation in follow‐up studies.

Although NaB inhibited tumour growth in the C26 cachexia model, it had no significant effect on tumour growth in the LLC model, indicating that its protective effects against cachexia are not dependent on its anticancer properties (Figure S4l,S4m). In other studies, NaB modulates muscle satellite cells by altering DNA methylation, reducing antibiotic‐induced activation and loss during regeneration [36, 37]. It also enhances muscle mass and function in sarcopenia by inhibiting FoxO3a/Atrogin‐1 and activating mTOR [38]. These insights support butyrate's role in mitigating cancer‐related cachexia muscle atrophy.

Prebiotics' health benefits have been widely studied [12]. Bindels et al. showed that pectin oligosaccharides and inulin protected leukaemia mice's fat and muscle in a Bcr‐Abl‐transfected proB lymphocyte model [39], but the specific mechanisms were unclear. Our research provided an extension to Bindels's findings. While inulin is reported to have antitumor effects [40], our data did not show significant change, likely due to their heterogeneity (Figure S7i). In this process, inulin‐mediated alleviation of cachexia may be related to *Muribaculaceae* and its butyrate metabolism improvement [9]. Inulin modulated the *Muribaculaceae* family and positively impacted other bacterial communities, like *Prevotellaceae*, *Bacteroidales* and *Lachnospirales*, which may synergize to combat muscle atrophy.

Our study advances understanding of the gut microbiome–muscle health nexus but has limitations. The gut microbiome–muscle interaction mechanisms require further investigation of molecular pathways. Although dietary inulin increased the abundance of specific commensal bacteria, such as the *Muribaculaceae* family, its clinical efficacy needs validation. Moreover, prebiotic metabolic effects likely involve complex microbial networks, not just individual species. Clarifying these interactions is crucial for maintaining gut homeostasis and health.

In conclusion, our findings reveal that a high‐fibre diet enhances gut microbiota stability, enriches beneficial bacteria such as *Muribaculaceae* and *Muribaculum intestinale*, and boosts butyrate levels, potentially mitigating inflammation and metabolic disorders in mouse muscle and improving cancer cachexia.

## Conflicts of Interest

The authors declare no conflicts of interest.

## Supporting Information

Additional supporting information can be found online in the Supporting Information section at the end of the article.

## Supporting information


**Figure S1:** **Abnormal gut microbiota distribution and reduced *Muribaculaceae* and *Muribaculum intestinale* abundance in cachexia mouse models. (a)‐(f)** C26 cancer cachexia model (*n* = 6) **(a)** Statistical analysis of α‐diversity indices (Chao1, Shannon, ACE, Simpson) across different samples. **(b)** Tax4fun2 analysis. **(c)‐(f)** The relative abundance normalized to Control Mean. **(g)‐(k)** LLC cancer cachexia model (*n* = 6) **(g)** Statistical analysis of α‐diversity indices (Chao1, Shannon, ACE, Simpson) across different samples. **(h)** PCoA analysis illustrating the differences in gut microbiota between normal and cachectic mice. The purple and orange shadows represent clustering within the groups. **(i)** Stacked bar‐plot of the relative abundances at the phylum level. **(j)** The differences in gut microbiota abundance between the normal and cachectic groups using linear discriminant analysis effect size (LEfSe) analysis. **(k)** Tax4fun2 analysis. The data are represented as the mean ± SEM. Two‐tailed unpaired Student's *t*‐tests were used. Statistical significance: ns means no significance; *p < 0.05; **p < 0.01; ****p < 0.0001.
**Figure S2.** ***Muribaculum** **intestinale*** **supplementation** **mitigates** **cancer** **cachexia** **in** **C26** **and** **LLC** **mice**.**(a)** Body weight change after tumour implantation. **(b)** Photos of muscle in each group showing the effect of *Muribaculum intestinale* supplementation on muscle. **(c)** Quadriceps showing the changes in *Muribaculum intestinale* supplemented mice (*n* = 5). **(d)** Tumour weight (*n* = 5). **(e)** The statistical analysis of Figure 2d. **(f)** The statistical analysis of Figure 2e. **(g)** Weight of lean mass showing the changes in *Muribaculum intestinale* supplemented mice (*n* = 5). **(h)** Photos of muscle in each group showing the effect of *Muribaculum intestinale* supplementation on muscle. **(i)** Grip strength of mice in each group before the end of the experiment (*n* = 5). **(j)** The ratio of tumour weight (*n* = 5). **(k)** H&E staining of quadriceps muscle showing the effect of *Muribaculum intestinale* supplementation on muscle, the statistical analysis is shown on the right panel. The scale bar represents 100 μm. **(l)** Relative mRNA expression of *Fbxo32* and *Murf1* in muscle from each group (*n* = 5). **(m)** The corresponding protein expression of Atrogin‐1, MuRF1, and p‐STAT3 in the muscle of *Muribaculum intestinale* supplemented mice. **(n)** The abundance of the *Muribaculum intestinale* after supplementation (*n* = 3). The data are presented as the mean ± SEM. Two‐tailed unpaired Student's *t*‐tests and one‐way ANOVA were used. Statistical significance: ns means no significance; *p < 0.05; **p < 0.01; ***p < 0.001; ****p < 0.0001.
**Figure S3: Reduced levels of SCFAs in the faeces of cachexia mice, positively correlated with the abundance of *Muribaculaceae* and *Muribaculum* intestinale. (a)** Quantification of SCFAs in the faeces of C26 cachexia mice. **(b‐c)** PCA of muscle metabolomics illustrating the differences of metabolites between control and cachectic mice. **(d)** Heatmap of differential metabolites in positive ion mode. (e) Heatmap of differential metabolites in negative ion mode. **(f)** The correlation heatmap at the phylum level. **(g)** The correlation heatmap at the class level. **(h)** The correlation heatmap at the order level. The data represent mean ± SEM. Two‐tailed unpaired Student's *t*‐test was used (*n* = 6). Statistical significance: ns means no significance; *p < 0.05; **p < 0.01; ***p < 0.001.
**Figure S4: Sodium butyrate supplementation alleviates weight loss and suppresses muscle degradation in cachexia mice. (a)‐(h)** C26 cancer cachexia model: **(a)** Body weight change after tumour implantation. **(b)** Weight change ratio of cachectic mice with sodium butyrate (NaB) treatment on day 8 and day 13 post‐tumour inoculation. **(c)** Body weight changes in control and cachexia mice treated with NaB on day 13 post‐tumour inoculation (*n* = 5). **(d)** Photos of muscle and eWAT in each group showing the effect of NaB supplementation on muscle and WAT. **(e)** The weight of the tumours. **(f)** The statistical analysis of Figure 4d. **(g)** H&E staining of eWAT showing the effect of NaB supplementation, the statistical analysis is shown on the right panel. The scale bar represents 100 μm. **(h)‐(l)** LLC cancer cachexia model: **(h)** H&E staining of quadriceps muscle of NaB supplementation, the statistical analysis is shown on the right panel. The scale bar represents 100 μm. **(i)‐(j)** mRNA levels of *Fbxo32* **(i)** and *Murf1* **(j)** in muscle from each group **(*n* = 5). (k)** The weight of the tumours. **(l)** The ratio of tumour weight. The data are presented as the mean ± SEM. Two‐tailed unpaired Student's *t*‐tests and one‐way ANOVA were used. Statistical significance: ns means no significance, *p < 0.05; **p < 0.01;****p < 0.0001.
**Figure S5: The muscle RNA‐seq demonstrates that sodium butyrate treatment can affect the expression levels of multiple genes in cachexia skeletal muscle samples. (a)** Principal component analysis (PCA) showing the differences in gene expression with cachectic mice supplemented with butyrate (NaB) (*n* = 3). **(b)** Heatmap in each group showing the difference in three groups. **(c)** Volcano plot showing the significantly changed genes in cachectic mice; the orange are upregulated genes in the C26 group, and the purple are the downregulated genes in the group. **(d)** KEGG analysis showing the most featured pathway enrichment in C26 and NC groups (*n* = 3). **(e)‐(g)** GO enrichment analysis showing significant enrichment in biological processes **(e)**, cellular components **(f)**, and molecular functions **(g)** with cachectic mice supplemented with butyrate. **(h)** GSEA of TNF‐α‐signalling, Inflammatory Response, IL‐6‐JAK‐STAT3 signalling, and myogenesis in C26 and NC groups. **(i)** Mitophagy‐related genes in each group. The data are presented as the mean ± SEM. One‐way ANOVA was used (*n* = 3). Statistical significance: *p < 0.05, **p < 0.01
**Figure S6: The mechanism by which sodium butyrate and *Muribaculum* *intestinale* treatment improves cachexia‐related muscle atrophy. (a)‐(d)** Inflammatory cytokine levels in the plasma of C26 mice: **(a)** TNF‐α, **(b)** IL‐6, **(c)** IL‐1β, and **(d)** MCP‐1 levels in each group (*n* = 5). **(e)**‐**(h)** Inflammatory cytokine levels in the plasma of LLC cachexia mice: **(e)** TNF‐α, **(f)** IL‐6, **(g)** IL‐1β, **(h)** MCP‐1 levels in each group (*n* = 4–5). **(i)** Relative mRNA levels of *Pdk4* in each group of the LLC cachexia mice **(*n* = 5)**. **(j)** Relative mRNA levels of *Pdk4* in each group of the C26 cachexia mice with *Muribaculum intestinale* supplementation (*n* = 5). The data are presented as the mean ± SEM. One‐way ANOVA was used. Statistical significance: ns means no significance, *p < 0.05, **p < 0.01, ***p < 0.001, ****p < 0.0001.
**Figure S7: Supplementation with inulin restored intestinal microbiota stability, enhanced *Muribaculaceae* abundance, and ameliorated cancer cachexia. (a)** Schematic of inulin supplementation in cachexia mice. **(b)** 2D PCoA Plot of 16S rRNA gene amplicon sequencing in each group. **(c)‐(f)** Stacked bar plots illustrating the relative abundances of microbial communities at various taxonomic levels: **(c)** phylum, **(d)** class, **(e)** order, and **(f)** family. **(g)** Body weight change after tumour implantation (*n* = 6). **(h)** Weight of quadriceps muscle showing the changes in inulin‐supplemented mice. **(i)** Tumour weight. **(j)** eWAT weight. **(k)** Heart weight. **(l)** Photographs of muscle and eWAT in each group showing the effect of inulin supplementation on muscle and eWAT (*n* = 6). **(m)** H&E staining of eWAT, and the statistical analysis is shown on the right panel. The scale bar represents 100 μm. The data are presented as the mean ± SEM. Two‐tailed unpaired Student's *t*‐tests and one‐way ANOVA were used (*n* = 6). Statistical significance: ns means no significance; *p < 0.05; ***p < 0.001; ****p < 0.0001.
**Figure S8: Schematic illustration of the mechanism.** The proposed mechanism diagram illustrates how ***Muribaculum intestinale*,** sodium butyrate and inulin alleviate cachexia symptoms. In this study, the abundance of *Muribaculaceae* and *Muribaculum intestinale* are both reduced and exhibited a significant positive correlation with SCFA butyrate. Inulin or MI supplementation increased these bacteria, ameliorating cachexia. Sodium butyrate reduced muscle wasting by decreasing autophagy and inflammation, inhibiting PDK4, and increasing ATP, suggesting a gut‐muscle connection in cachexia. These findings underscore the potential of microbiota‐targeted interventions in managing cancer cachexia and highlight the intricate interplay between gut microbiota and skeletal muscle health.
**Table S1:** List of primary and secondary antibodies.
**Table S2:** List of primers used for qPCR analyses.
**Table S3:** Compositions of different diets.
