# Supplementary material for: Restoring Muribaculum intestinale–Derived Butyrate Mitigates Skeletal Muscle Loss in Cancer Cachexia
Source: J Cachexia Sarcopenia Muscle. 2025 Nov 26;16(6):e70140. doi: 10.1002/jcsm.70140 (PMC12657623; doi:10.1002/jcsm.70140)
Supplement: Supplementary file 1 — Figure S1: Abnormal gut microbiota distribution and reduced Muribaculaceae and Muribaculum intestinale abundance in cachexia mouse models. (a)‐(f) C26 cancer cachexia model (n = 6) (a) Statistical analysis of α‐diversity indices (Chao1, Shannon, ACE, Simpson) across different samples. (b) Tax4fun2 analysis. (c)‐(f) The relative abundance normalized to Control Mean. (g)‐(k) LLC cancer cachexia model (n = 6) (g) Statistical analysis of α‐diversity indices (Chao1, Shannon, ACE, Simpson) across different samples. (h) PCoA analysis illustrating the differences in gut microbiota between normal and cachectic mice. The purple and orange shadows represent clustering within the groups. (i) Stacked bar‐plot of the relative abundances at the phylum level. (j) The differences in gut microbiota abundance between the normal and cachectic groups using linear discriminant analysis effect size (LEfSe) analysis. (k) Tax4fun2 analysis. The data are represented as the mean ± SEM. Two‐tailed unpaired Student's t‐tests were used. Statistical significance: ns means no significance; *p < 0.05; **p < 0.01; ****p < 0.0001. Figure S2. Muribaculum intestinale supplementation mitigates cancer cachexia in C26 and LLC mice.(a) Body weight change after tumour implantation. (b) Photos of muscle in each group showing the effect of Muribaculum intestinale supplementation on muscle. (c) Quadriceps showing the changes in Muribaculum intestinale supplemented mice (n = 5). (d) Tumour weight (n = 5). (e) The statistical analysis of Figure 2d. (f) The statistical analysis of Figure 2e. (g) Weight of lean mass showing the changes in Muribaculum intestinale supplemented mice (n = 5). (h) Photos of muscle in each group showing the effect of Muribaculum intestinale supplementation on muscle. (i) Grip strength of mice in each group before the end of the experiment (n = 5). (j) The ratio of tumour weight (n = 5). (k) H&E staining of quadriceps muscle showing the effect of Muribaculum intestinale supplementation o [file JCSM-16-e70140-s001.docx]

**Supplementary Figure**

**Li et al. 2025**

**Figure S1**


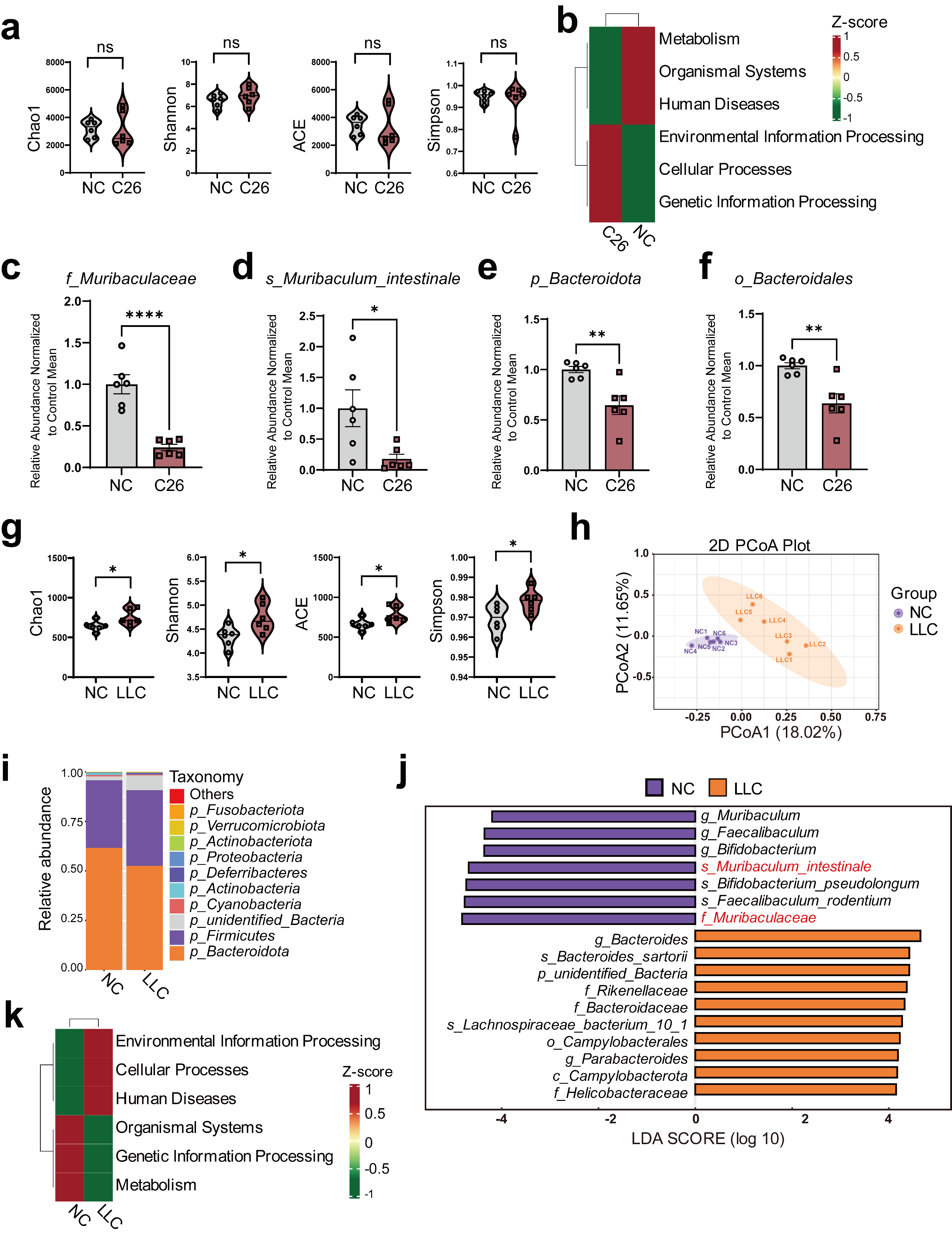


**Figure S1. Abnormal gut microbiota distribution and reduced *Muribaculaceae* and *Muribaculum intestinale* abundance in cachexia mouse models.**

**(a)-(f)** C26 cancer cachexia model (*n*=6) **(a)** Statistical analysis of α-diversity indices (Chao1, Shannon, ACE, Simpson) across different samples. **(b)** Tax4fun2 analysis. **(c)-(f)** The relative abundance normalized to Control Mean. **(g)-(k)** LLC cancer cachexia model (*n*=6) **(g)** Statistical analysis of α-diversity indices (Chao1, Shannon, ACE, Simpson) across different samples. **(h)** PCoA analysis illustrating the differences in gut microbiota between normal and cachectic mice. The purple and orange shadows represent clustering within the groups. **(i)** Stacked bar-plot of the relative abundances at the phylum level. **(j)** The differences in gut microbiota abundance between the normal and cachectic groups using linear discriminant analysis effect size (LEfSe) analysis. **(k)** Tax4fun2 analysis. The data are represented as the mean ± SEM. Two-tailed unpaired Student’s *t*-tests were used. Statistical significance: ns means no significance; **p* < 0.05；***p* < 0.01; *****p* < 0.0001.

Figure S2 **
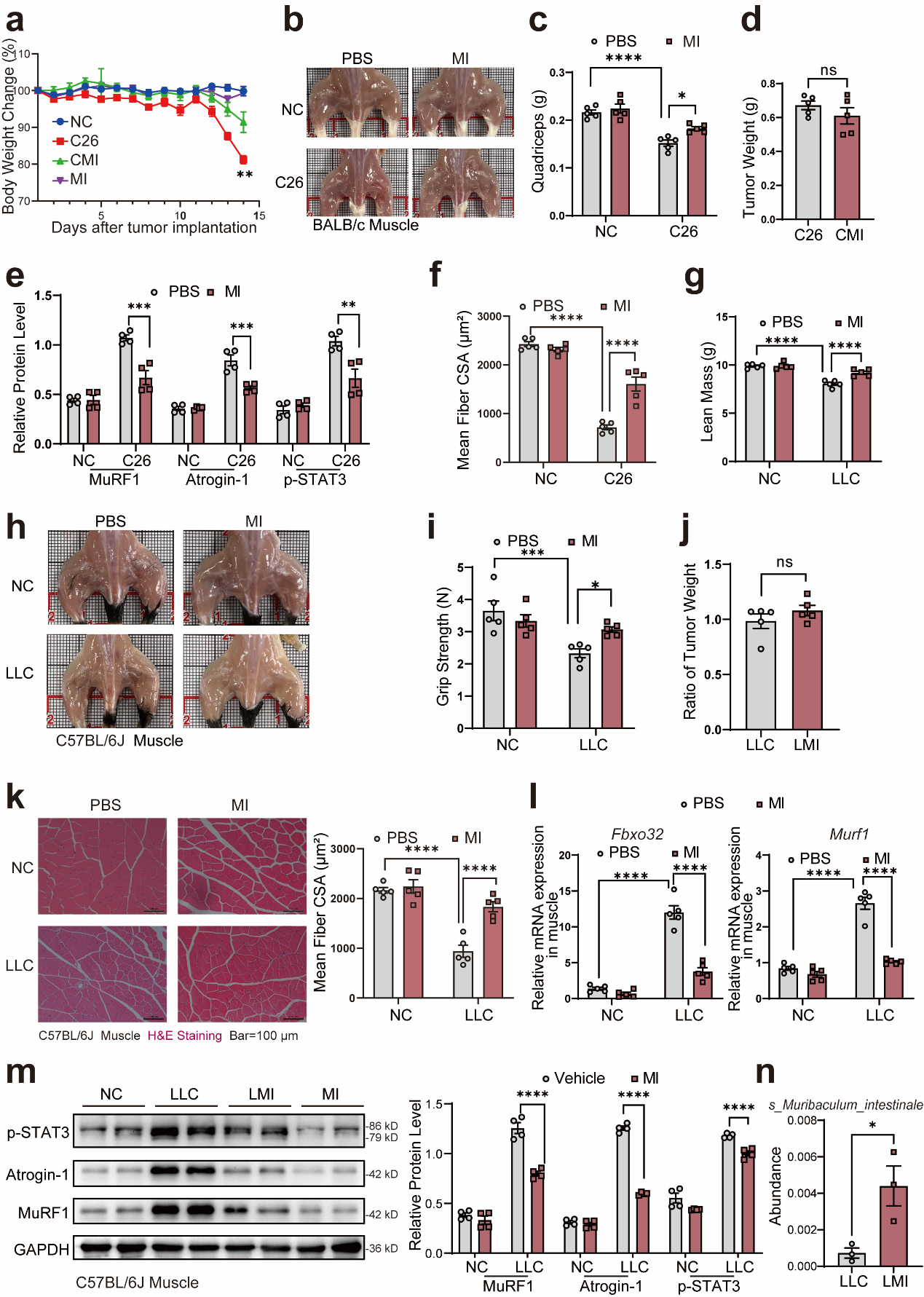
**

**Figure S2****.** ***Muribaculum intestinale* supplementation mitigates cancer cachexia in C26 and LLC mice.**

**(a)** Body weight change after tumor implantation. **(b)** Photos of muscle in each group showing the effect of *Muribaculum intestinale* supplementation on muscle. **(c)** Quadriceps showing the changes in *Muribaculum intestinale* supplemented mice (*n*=5). **(d)** Tumor weight (*n*=5). **(e)** The statistical analysis of Figure 2d. **(f)** The statistical analysis of Figure 2e. **(g)** Weight of lean mass showing the changes in *Muribaculum intestinale* supplemented mice (*n*=5). **(h)** Photos of muscle in each group showing the effect of *Muribaculum intestinale* supplementation on muscle. **(i)** Grip strength of mice in each group before the end of the experiment (*n*=5). **(j)** The ratio of tumor weight (*n*=5). **(k)** H&E staining of quadriceps muscle showing the effect of *Muribaculum intestinale* supplementation on muscle, the statistical analysis is shown on the right panel. The scale bar represents 100 μm. **(l)** Relative mRNA expression of *Fbxo32* and *Murf1* in muscle from each group (*n*=5). **(m)** The corresponding protein expression of Atrogin-1, MuRF1, and p-STAT3 in the muscle of *Muribaculum intestinale* supplemented mice. **(n)** The abundance of the *Muribaculum intestinale* after supplementation (*n*=3). The data are presented as the mean ± SEM. Two-tailed unpaired Student’s *t*-tests and one-way ANOVA were used. Statistical significance: ns means no significance; **p* < 0.05; ***p* < 0.01; ****p* < 0.001; *****p* < 0.0001.

**Figure S3**

**
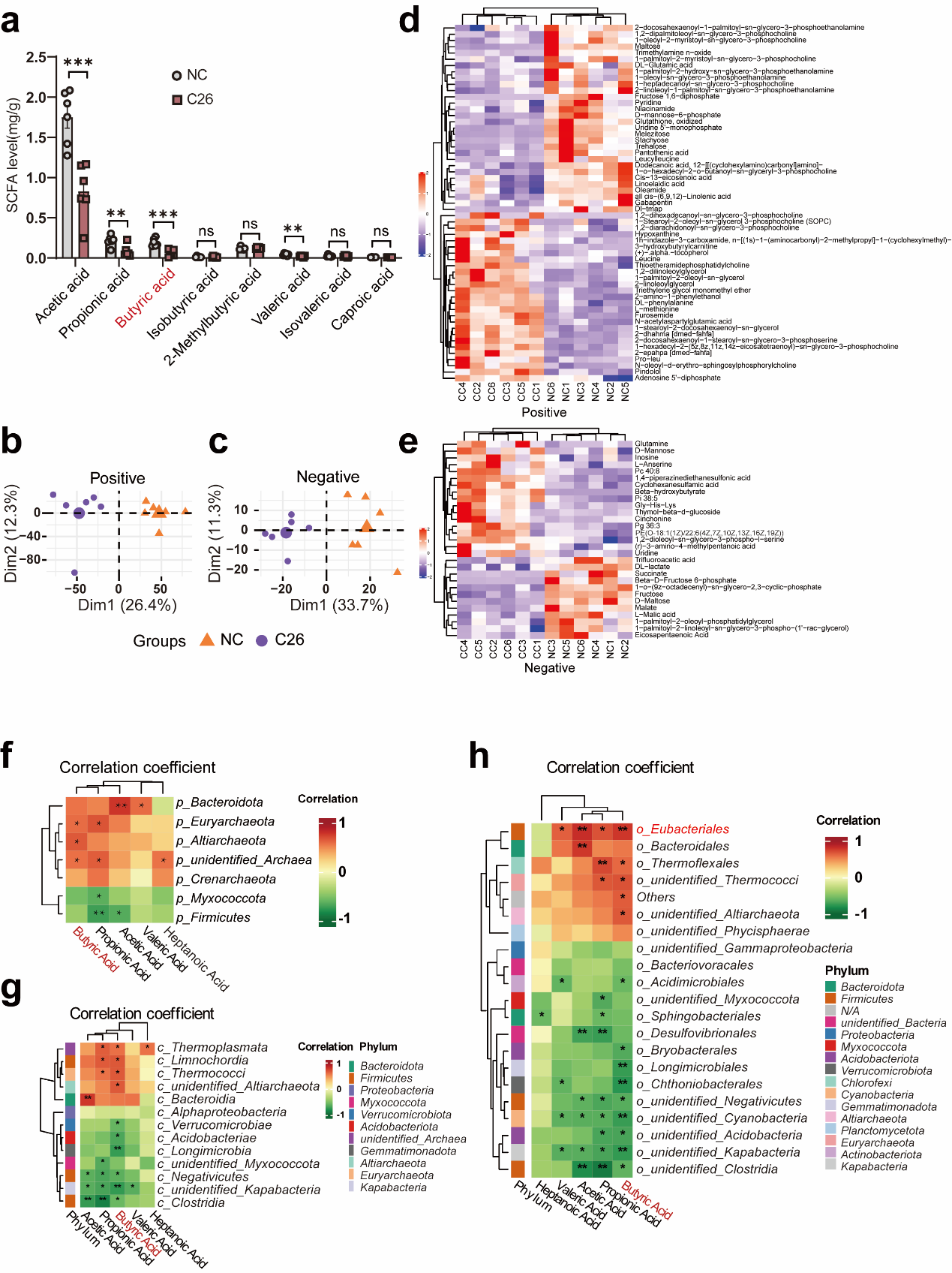
**

**Figure S3. Reduced levels of SCFAs in the feces of cachectic mice, positively correlated with the abundance of *Muribaculaceae* and *Muribaculum intestinale*.**

**(a)** Quantification of SCFAs in the feces of C26 cachexia mice. **(b-c)** PCA of muscle metabolomics illustrating the differences of metabolites between control and cachectic mice. **(d)** Heatmap of differential metabolites in positive ion mode. (e) Heatmap of differential metabolites in negative ion mode. **(f)** The correlation heatmap at the phylum level. **(g)** The correlation heatmap at the class level. **(h)** The correlation heatmap at the order level. The data represent mean ± SEM. Two-tailed unpaired Student’s *t*-test was used (*n*=6). Statistical significance: ns means no significance; **p* < 0.05; ***p* < 0.01; ****p* < 0.001.

**Figure S4**


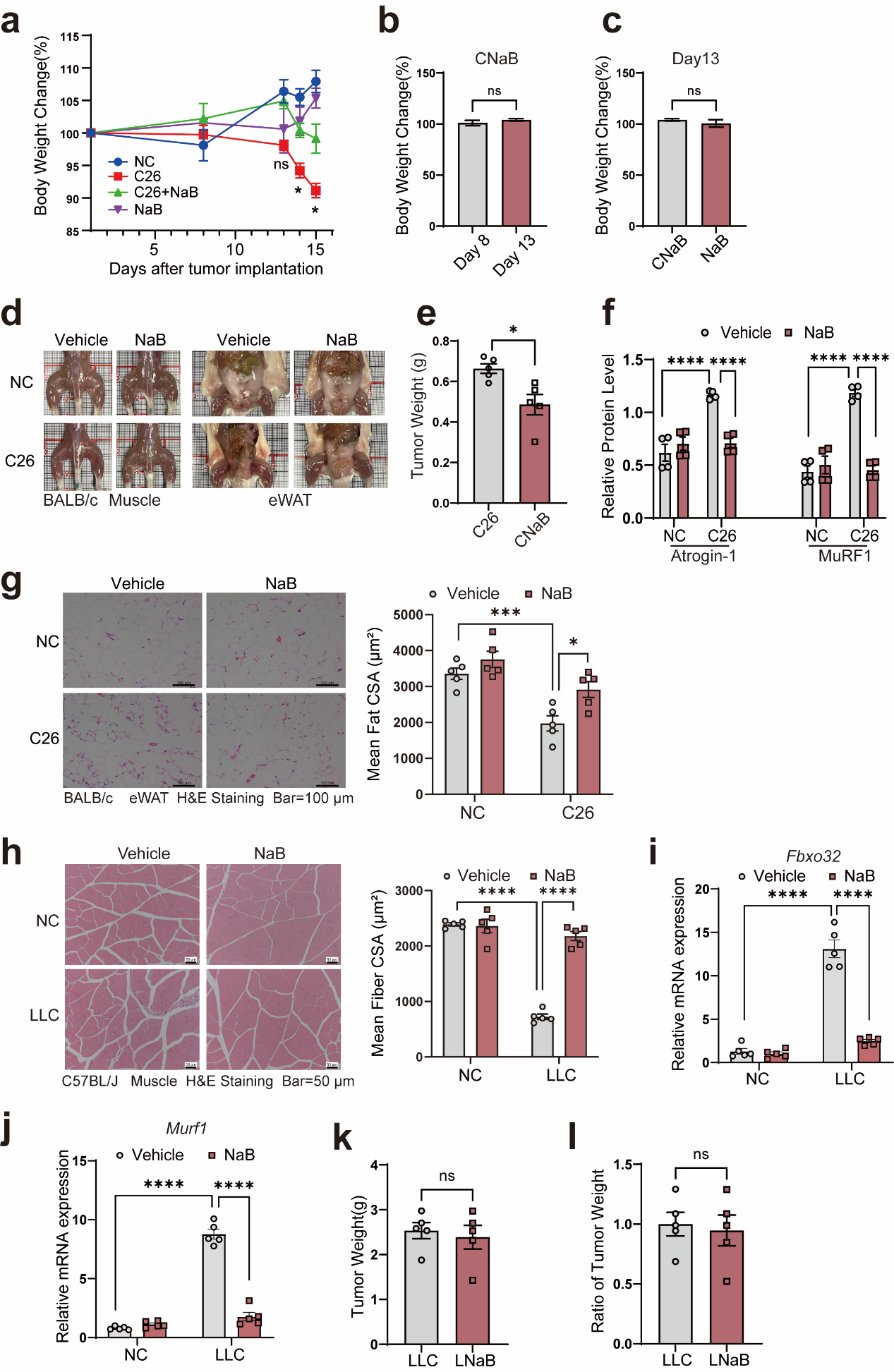


**Figure S4. Sodium butyrate supplementation alleviates weight loss and suppresses muscle degradation in cachexia mice.**

**(a)-(h)** C26 cancer cachexia model: **(a)** Body weight change after tumor implantation. **(b)** Weight change ratio of cachectic mice with sodium butyrate(NaB) treatment on day 8 and day 13 post-tumor inoculation. **(c)** Body weight changes in control and cachexia mice treated with NaB on day 13 post-tumor inoculation (*n*=5). **(d)** Photos of muscle and eWAT in each group showing the effect of NaB supplementation on muscle and WAT. **(e)** The weight of the tumors. **(f)** The statistical analysis of Figure 4d. **(g)** H&E staining of eWAT showing the effect of NaB supplementation, the statistical analysis is shown on the right panel. The scale bar represents 100 μm. **(h)-(l)** LLC cancer cachexia model: **(h)** H&E staining of quadriceps muscle of NaB supplementation, the statistical analysis is shown on the right panel. the scale bar represents 100 μm. **(i)-(j)** mRNA levels of *Fbxo32* **(i)** and *Murf1* **(j)** in muscle from each group **(*n*=5). (k)** The weight of the tumors. **(l)** The ratio of tumor weight. The data are presented as the mean ± SEM. Two-tailed unpaired Student’s *t*-tests and one-way ANOVA were used. Statistical significance: ns means no significance, **p* < 0.05; ***p* < 0.01;*****p* < 0.0001.

**Figure S5**

**
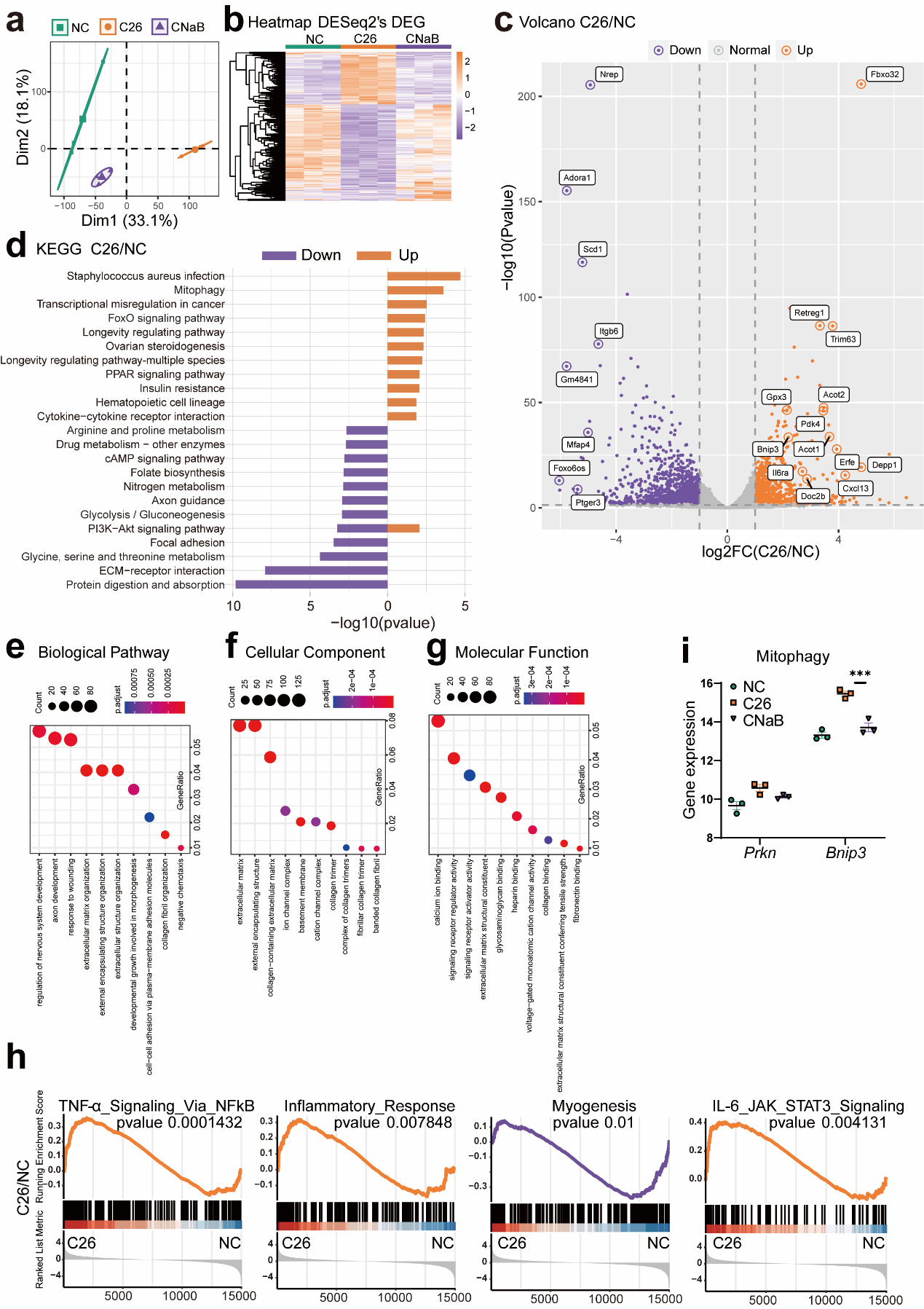
**

**Figure S5. The muscle RNA-seq demonstrates that sodium butyrate treatment can affect the expression levels of multiple genes in cachexia skeletal muscle samples.**

**(a)** Principal component analysis (PCA) showing the differences in gene expression with cachectic mice supplemented with butyrate (NaB) (*n*=3). **(b)** Heatmap in each group showing the difference in three groups. **(c)** Volcano plot showing the significantly changed genes in cachectic mice; the orange are upregulated genes in the C26 group, and the purple are the downregulated genes in the group. **(d)** KEGG analysis showing the most featured pathway enrichment in C26 and NC groups (*n*=3). **(e)-(g)** GO enrichment analysis showing significant enrichment in biological processes **(e)**, cellular components **(f)**, and molecular functions **(g)** with cachectic mice supplemented with butyrate. **(h)** GSEA of TNF-α-signaling, Inflammatory Response, IL-6-JAK-STAT3 signaling, and myogenesis in C26 and NC groups. **(i)** Mitophagy-related genes in each group. The data are presented as the mean ± SEM. One-way ANOVA was used (*n*=3). Statistical significance: **p* < 0.05, ***p* < 0.01.

**Figure S6**


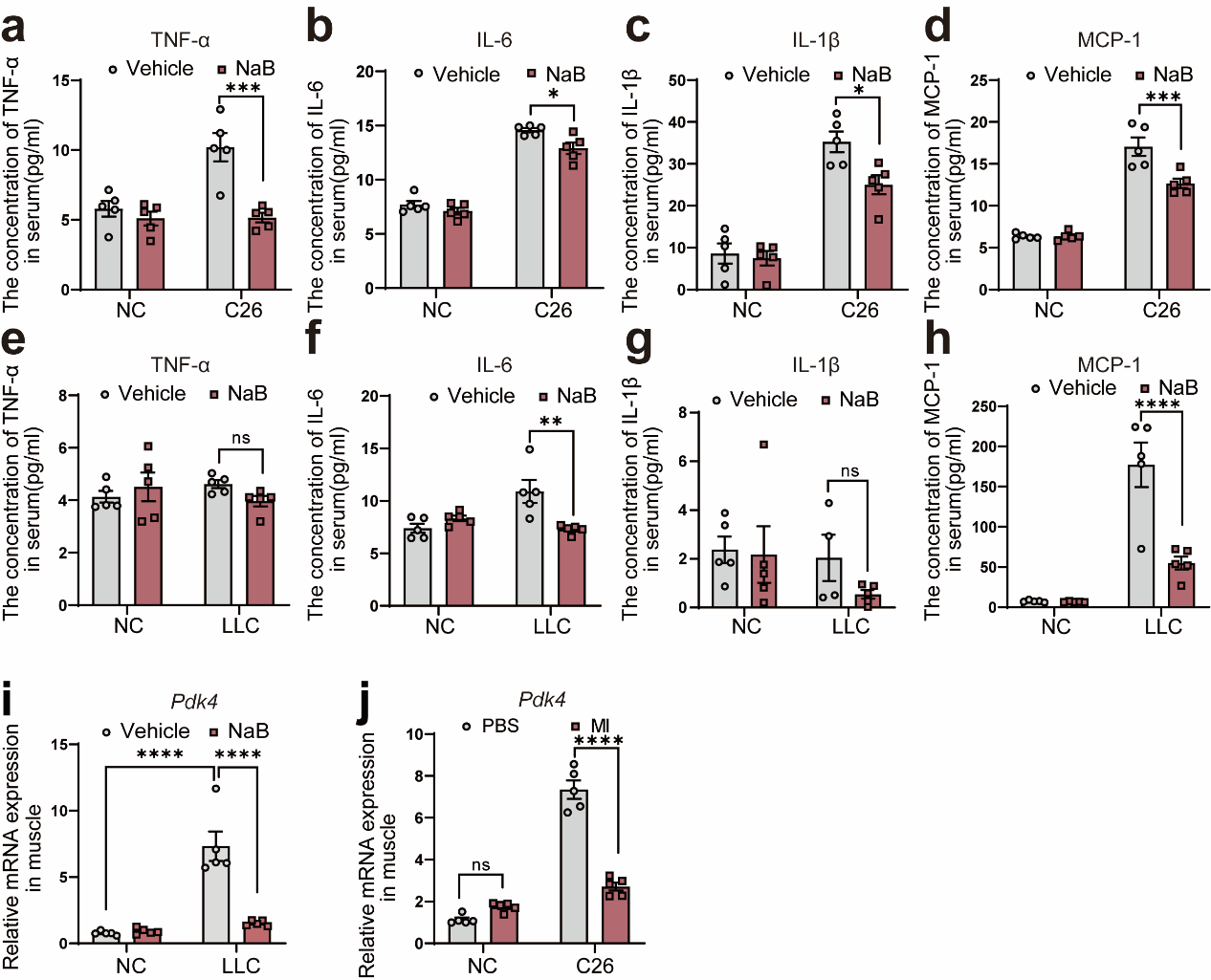


**Figure S6. The mechanism by which sodium butyrate and** ***Muribaculum intestinale* treatment improves cachexia-related muscle atrophy.**

**(a)-(d)** Inflammatory cytokine levels in the plasma of C26 mice: **(a)** TNF-α, **(b)** IL-6, **(c)** IL-1β, and **(d)** MCP-1 levels in each group (*n*=5). **(e)**-**(h)** Inflammatory cytokine levels in the plasma of LLC cachexia mice: **(e)** TNF-α, **(f)** IL-6, **(g)** IL-1β, **(h)** MCP-1 levels in each group (*n*=4-5). **(i)** Relative mRNA levels of *Pdk4* in each group of the LLC cachexia mice (*n*=5). **(j)** Relative mRNA levels of *Pdk4* in each group of the C26 cachexia mice with *Muribaculum intestinale* supplementation (*n*=5). The data are presented as the mean ± SEM. One-way ANOVA was used. Statistical significance: ns means no significance, **p* < 0.05, ***p* < 0.01, ****p* < 0.001, *****p* < 0.0001.

**Figure S7**

**
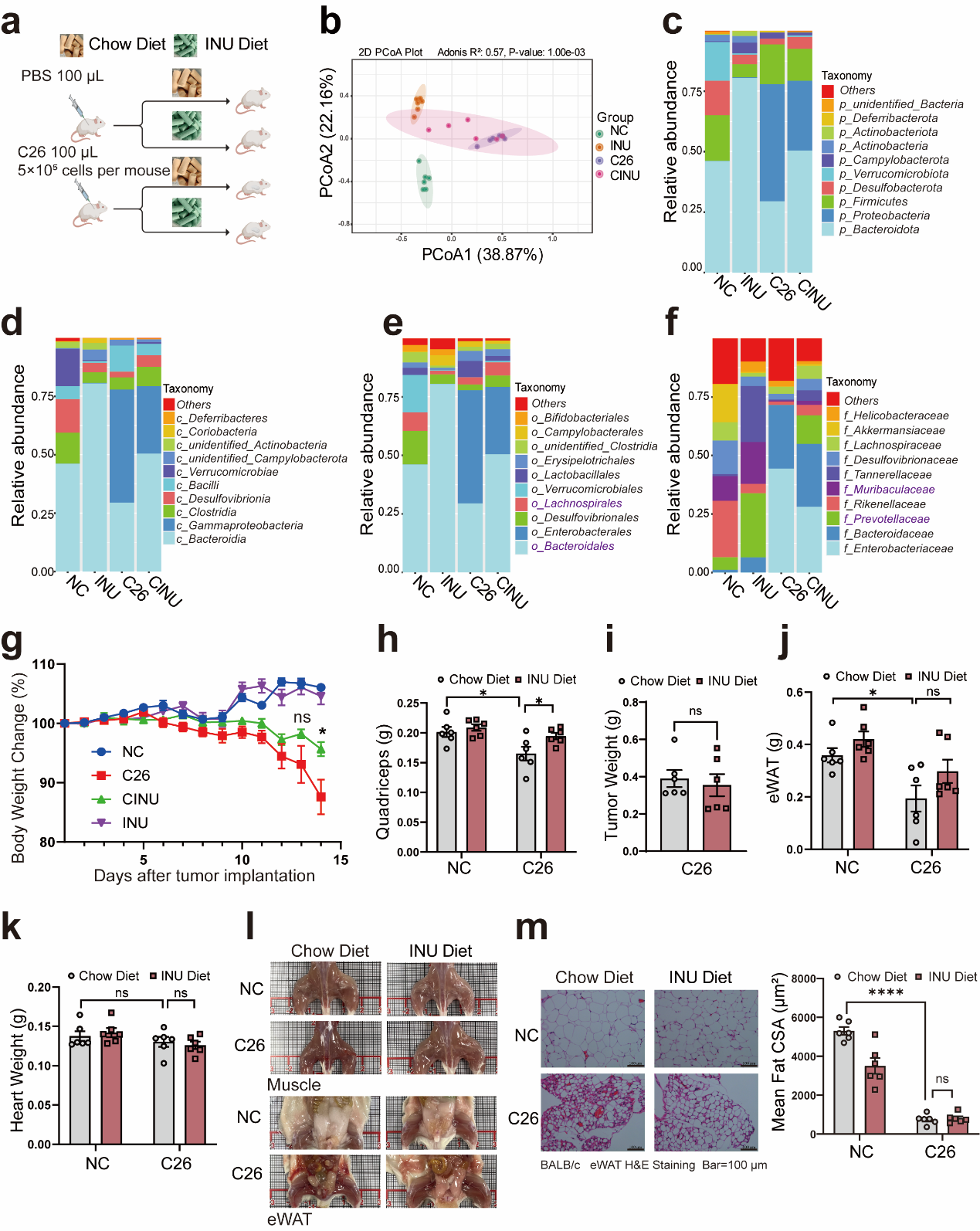
**

**Figure S7. Supplementation with inulin restored intestinal microbiota stability, enhanced *Muribaculaceae* abundance, and ameliorated cancer cachexia.**

**(a)** Schematic of inulin supplementation in cachexia mice. **(b)** 2D PCoA Plot of 16S rRNA gene amplicon sequencing in each group. **(c)-(f)** Stacked bar plots illustrating the relative abundances of microbial communities at various taxonomic levels: **(c)** phylum, **(d)** class, **(e)** order, and **(f)** family. **(g)** Body weight change after tumor implantation (*n*=6). **(h)** Weight of quadriceps muscle showing the changes in inulin-supplemented mice. **(i)** Tumor weight. **(j)** eWAT weight. **(k)** Heart weight. **(l)** Photographs of muscle and eWAT in each group showing the effect of inulin supplementation on muscle and eWAT (*n*=6). **(m)** H&E staining of eWAT, and the statistical analysis is shown on the right panel. The scale bar represents 100 μm. The data are presented as the mean ± SEM. Two-tailed unpaired Student’s *t*-tests and one-way ANOVA were used (*n*=6). Statistical significance: ns means no significance; **p* < 0.05; ****p* < 0.001; *****p* < 0.0001.

Figure S8.


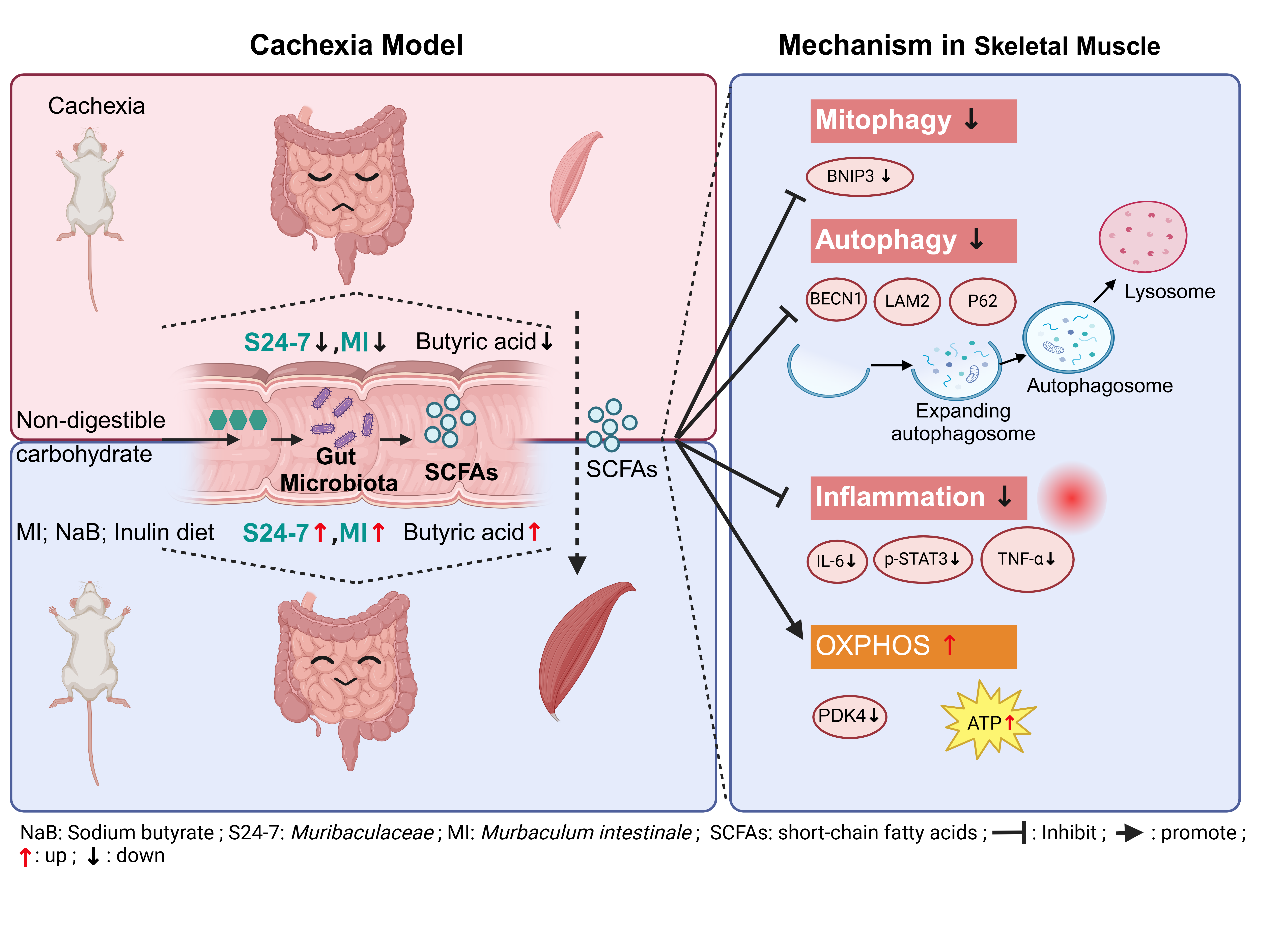


**Figure S8 Schematic illustration of the mechanism**

The proposed mechanism diagram illustrates how *Muribaculum intestinale*, sodium butyrate and inulin alleviate cachexia symptoms. In this study, the abundance of *Muribaculaceae* and *Muribaculum intestinale* are both reduced and exhibited a significant positive correlation with SCFA butyrate. Inulin or MI supplementation increased these bacteria, ameliorating cachexia. Sodium butyrate reduced muscle wasting by decreasing autophagy and inflammation, inhibiting PDK4, and increasing ATP, suggesting a gut-muscle connection in cachexia. These findings underscore the potential of microbiota-targeted interventions in managing cancer cachexia and highlight the intricate interplay between gut microbiota and skeletal muscle health.

**Supplementary Tables**

**Supplementary Table 1**

Supplementary Table 1. List of primary and secondary antibodies.

| Antibody | Customer | Catlog |
| --- | --- | --- |
| Atrogin-1 | Santa Cruz | sc-166806 |
| MuRF1 | Santa Cruz | sc-398608 |
| LC3 | Proteintech | 14600-1-AP |
| p-STAT3 | Cell Singnaling Technology | 9145S |
| α-Actin | Abclonal | A2235 |
| β-Tubulin | Abclonal | AC021 |
| Goat Anti-Rabbit IgG (H+L) HRP | Bioworld | BS13278 |
| Goat Anti-Mouse IgG (H+L) HRP | Bioworld | BS12478 |

**Supplementary Table 2**

Supplementary Table 2. List of primers used for qPCR analyses.

| **Genes** | **Forward primer (5’-3’)** | **Reverse primer (5’-3’)** |
| --- | --- | --- |
| m-*Murf1* | TACCAAGCCTGTGGTCATCCTG | ACGGAAACGACCTCCAGACATG |
| m-*Fbxo32* | CTTCTCGACTGCCATCCTGGAT | TCTTTTGGGCGATGCCACTCAG |
| m-*Pdk4* | TCGACCCAAACTGTGATGTG | TCGAAGAGCATGTGGTGAAG |
| m-*Becn1* | CAGCCTCTGAAACTGGACACGA | CTCTCCTGAGTTAGCCTCTTCC |
| m-*Gabarap* | CAAAGAGGAGCATCCGTTCGAG | TTGTCCAGGTCTCCTATCCGAG |
| m-*Lamp2* | GAGCAGGTGCTTTCTGTGTCTAG | GCCTGAAAGACCAGCACCAACT |
| m-*Map1lc3b* | GTCCTGGACAAGACCAAGTTCC | CCATTCACCAGGAGGAAGAAGG |
| m-*Sqstm1* | GCTCTTCGGAAGTCAGCAAACC | GCAGTTTCCCGACTCCATCTGT |
| m-*Bnip3* | GTCGCCTGGCCTCAGAAC | CCCATTGCCATTGCTGAAGT |
| m-*Actb* | GCTGTATTCCCCTCCATCGT | AGGTGTGGTGCCAGATCTTC |

**Supplementary Table 3**

Supplementary Table 3. Compositions of different diets

| **Ingredient** | **Chow Diet** | **Inulin Diet** |
| --- | --- | --- |
| **Ingredient** | **gm** | **gm** |
| Casein (g) | 199.20 | 180.70 |
| Corn starch (g) | 447.50 | 406.10 |
| Dextrin (g) | 117.00 | 73.10 |
| Sucrose (g) | 66.50 | 60.30 |
| Bean oil (g) | 24.90 | 22.60 |
| Lard (g) | 44.80 | 40.70 |
| Cellulose (g) | 49.80 | 45.20 |
| Mineral substance (g) | 34.90 | 31.60 |
| Vitamin (g) | 10.00 | 9.00 |
| L-Cystine (g) | 3.00 | 2.70 |
| Choline chloride (g) | 2.50 | 2.30 |
| Inulin (g) | 0.00 | 125.80 |
| TBHQ (g) | 0.01 | 0.01 |
| Total (g) | 1000.00 | 1000.00 |
| **Nutrient Percentage** | **kcal%** | **kcal** |
| Protein | 19.40 | 19.40 |
| Carbohydrate | 63.90 | 63.90 |
| Fat | 16.70 | 16.70 |
| Total Calories | 100.00 | 100.00 |
| Caloric Density (kcal/g) | 3.70 | 3.40 |
| Inulin (g/kcal) | 0 | 0.037 |

**Supplementary methods**

***Muribaculum intestinale culture***

The type strain of *Muribaculum intestinale* (DSM 28989) was obtained from Mingzhoubio Co., Ltd. (Ningbo, China). Bacterial cultivation was performed in modified Carboxymethylcellulose sodium (CMC) medium under strictly anaerobic conditions [1, 2]. CMC Medium (5.75 g/L in distilled water) was autoclaved (115℃, 20 min) and supplemented with filter-sterilized vitamin K1 (5 μg/mL) and hemin (0.5 μg/mL) at 45℃ under nitrogen atmosphere. After transferring to an anaerobic chamber (37℃ preconditioned), the medium was inoculated with thawed glycerol stock and incubated anaerobically for 72 h at 37℃. Bacterial cells were collected by centrifugation (5,000 × g, 5 min) and washed twice with anaerobic PBS. The pellet was resuspended to achieve 10^9^ CFU/mL using McFarland standard calibration, with viability confirmed by plate counting.

***Animal models and expirements***

As for the inulin diet treatment, the BALB/c mice were randomly assigned to four groups: NC (control, chow diet, *n*=6), C26 (cachexia, chow diet, *n*=6), CINU (cachexia, inulin diet, *n*=6), and INU (control, chow diet, *n*=6). After C26 cells inoculation, the proportion of inulin in the diet gradually increased from 1:3 to 2:2 to 3:1 compared to the chow diet. The chow diet (CD) is inulin-free, while the inulin-containing diet (Inulin diet) has 125.8 g/kg of inulin (12.58% w/w) (see Table S3 of the Supplementary materials).

To prevent adverse effects from rapid dietary changes on gut microbiota, we adopted a three-stage progressive intervention design:

Days 1-5: A 1:3 mix of inulin diet and chow diet, delivering 31.45 g/kg of inulin.

Days 6-10: A 2:2 mix, increasing inulin intake to 62.9 g/kg.

Days 11-14: A final 3:1 mix, reaching 94.35 g/kg of inulin intake.

Throughout the experiment, the control group continuously received inulin - free basic feed. All feed types maintained identical macronutrient compositions.

After 14 days on the inulin diet, the mice were euthanized to collect tissue samples. At the endpoint of the experiment, serum, muscle, adipose tissue, and feces from the mice were collected. The weights of the epididymal white adipose tissue, single hind limb, quadriceps, and the lean mass of the mice were recorded. In the present study, lean mass generally refers to the weight of skeletal and muscular components in the trunk and limbs after removal of the head, tail, adipose tissue, and viscera in mice [3-6].

As for *Muribaculum intestinale* gavage treatment, twenty BALB/c mice were randomized into four groups: NC (control, PBS, *n*=5), C26 (cachexia, PBS, *n*=5), CMI (cachexia, *Muribaculum intestinale*, *n*=5), and MI (control, *Muribaculum intestinale*, *n*=5). After inoculation of C26 cells, the mice were orally gavaged daily with *Muribaculum intestinale* (10^8^ CFU/mouse/day) or PBS. Body weight and food intake were monitored daily, and after 14 days, the mice were euthanized for tissue sampling. Twenty C57BL/6J mice were subjected to the same protocol following LLC inoculation.

For sodium butyrate (NaB) treatment, twenty BALB/c mice were randomly assigned to four groups: NC (control, PBS, *n*=5), C26 (cachexia, PBS, *n*=5), CNaB (cachexia, NaB, *n*=5), and NaB (control, NaB, *n*=5). After C26 cells inoculation, mice were gavaged daily with NaB (50 mg/kg) or sterile water. Body weight was monitored, and mice were euthanized after 14 days for tissue sampling. Twenty C57BL/6J mice followed a similar protocol post-LLC inoculation.

This study was approved by the Ethics Committee for Animal Experiments of Nanjing University Medical School.

***Hematoxylin and eosin (H&E) staining***

Tissues were fixed in 4% paraformaldehyde for at least 24 hours, then paraffin-embedded, sectioned, and stained with hematoxylin and eosin (H&E) at Wuhan Sevier Biosciences Co., Ltd.. Observations were made under an optical microscope, and the cross-sectional areas (CSA) of muscle fibers and lipid droplets were statistically analyzed using ImageJ software.

***Immunofluorescence staining***

C2C12 cells were seeded on six-well plates, differentiated, and treated with drugs. Following careful removal of the culture medium, the cells were washed twice with PBS, fixed with 4% paraformaldehyde at room temperature for 15 minutes, and then washed again with PBS. Subsequently, 0.2% Triton X-100 (dissolved in PBS) was added after fixation. The cells were then incubated with primary antibody overnight at 4°C, with MyHC as the primary antibody (diluted 1:100 in antibody dilution buffer). The next day, after washing with PBS, the cells were incubated with the corresponding secondary antibody at room temperature in the dark for 1 h. Following washing with PBS, the coverslips were sealed with an anti-fluorescence quenching reagent containing DAPI. The fluorescence images were captured using FV3000 software and analyzed with ImageJ software. Scale calibration was performed by drawing a line over the scale bar, inputting the known distance (μm) under Analyze > Set Scale. For each myotube, three random positions along the longitudinal axis were measured for diameter, and the average value was calculated as the diameter of the myotube. Ten myotubes were randomly selected for statistical analysis [3-6].

***Protein extraction and Western blotting***

Muscle tissue or cells were lysed using RIPA lysis buffer containing protease and phosphatase inhibitors to extract tissue or cellular proteins. The protein concentration was determined using a BCA assay kit. SDS-PAGE was performed using 15-30 μg of total protein, followed by the transfer of proteins to a PVDF membrane after electrophoresis. After blocking with 5% BSA at room temperature for 1 h, the membrane was incubated with primary antibodies overnight at 4°C, followed by incubation with secondary antibodies at room temperature for 1 h. After incubation, the membrane was washed and then exposed using a chemiluminescent substrate kit. Image acquisition was performed using a Tanon chemiluminescence imaging analysis, and grayscale analysis was conducted using ImageJ software. All of the antibodies used were summarized in **Supplementary** **Table 1.**

***ATP content measurement***

The ATP content in muscle tissue was determined using an ATP assay kit following the steps outlined in the kit's instructions. Briefly, after tissue or cell lysis and centrifugation to collect the supernatant, an ATP standard curve was prepared. Subsequently, each sample was added to the detection solution and subjected to fluorescence detection using a chemiluminescence analyzer. To eliminate errors caused by differences in protein concentration, standardization procedures were performed after determining the protein concentration using the BCA method.

***Plasma inflammatory cytokine assessment***

Mouse blood was collected into heparinized tubes, centrifuged at 3000 rpm for 15 minutes to pellet cells, and plasma was stored at -80°C. Inflammatory cytokines (IL-1β, IL-6, TNF-α, IFN-γ, and MCP-1) were quantified using the ABplex Mouse 5-Plex Custom Panel following the manufacturer's protocol with ABclonal Technology Co., Ltd.

***16S rRNA gene* *amplicon sequencing and bioinformatics***

Collected mouse feces were immediately snap-frozen in liquid nitrogen and preserved at -80°C. Microbial genomic DNA was extracted using the QIAamp Fast DNA Stool Mini Kit (Qiagen) with bead-beating homogenization. Amplification of the V4 hypervariable region (515F/806R primers) and 2×250 bp paired-end sequencing were performed on the Illumina NovaSeq 6000 system. Bioinformatics processing followed QIIME2 standard pipeline [7]. Initial demultiplexing was performed by exact barcode matching, followed by rigorous quality control with fastp v0.22.0 to remove adapters (auto-detection), low-quality reads (Q20 < 50% of bases, polyG tails, or reads containing > 15 N bases), and short fragments (< 150 bp). High-quality paired-end reads were merged using FLASH v1.2.11 (max overlap: 250 bp; min overlap: 30 bp) [8]. Chimeric sequences were identified and removed via *de novo* detection with VSEARCH v2.22.1 against the SILVA 138.1 reference database [9]. Amplicon Sequence Variants (ASVs) were generated using DADA2 v1.26.0 in QIIME 2 (truncLen=c(240,200); taxonomic annotation was performed with a naive Bayes classifier trained on the SILVA 138.1 SSU rRNA dataset (confidence threshold: 0.8) [10]. Alpha diversity (defined as the diversity within an individual sample) was analyzed using the Chao1, Shannon, ACE, and Simpson indices. Functional profiles were predicted through Tax4Fun2 v1.1.5 using KEGG orthology (2021.01 release), normalized for 16S rRNA gene copy numbers. Differential ASVs were identified via LEfSe (LDA score >3.0, Wilcoxon *p* < 0.05 after FDR adjustment) [11]. The sequencing and subsequent analysis were performed by Wuhan MetWare Metabolic Biotechnology Co., Ltd.

***Short-chain fatty acid assessment***

Fecal samples (20 mg) were homogenized in 1 mL of 0.5% (v/v) phosphoric acid aqueous solution using a steel bead mill (3 min, 50 Hz). The homogenate was vortexed vigorously for 10 min, followed by ultrasonication for 5 min. After centrifugation at 12,000 r/min for 10 min (4℃), 100 μL of supernatant was transferred to a clean microcentrifuge tube and mixed with 500 μL methyl tert-butyl ether (MTBE) containing internal standards. The mixture was vortexed for 3 min, ultrasonicated for 5 min, and centrifuged at 12,000 r/min for 10 min (4℃).

The GC-MS/MS analysis was conducted under optimized conditions: helium carrier gas flow rate: 1.2 mL/min, split ratio: 5:1, injection volume: 1 μL. The oven temperature program initiated at 50℃ (1 min hold), followed by a ramp to 220℃ at 18℃/min (5 min final hold). The mass spectrometer operated in multiple reaction monitoring (MRM) mode with injector and transfer line temperatures maintained at 250℃ and 230℃, respectively [12]. The assessment was performed by Wuhan MetWare Metabolic Biotechnology Co., Ltd. using the Agilent 7890B-7000D GC-MS/MS platform.

***Untargeted metabolomics of muscle***

(1) Sample Preparation

Quadriceps muscle tissues (*n*=6) were homogenized in 200 μL ice-cold water using a ceramic bead disruptor. Metabolites were extracted with 800 μL chilled methanol/acetonitrile (1:1, v/v), followed by centrifugation (14,000 g, 15 min, 4°C). The supernatant was lyophilized and reconstituted in 100 μL acetonitrile/water (1:1, v/v) prior to LC-MS/MS analysis.

(2) LC-MS/MS Analysis

Chromatographic separation was performed on an Agilent 1290 UHPLC system coupled to an AB Sciex TripleTOF 6600 mass spectrometer. Two separation modes were employed:

HILIC mode: An ACQUITY UPLC BEH column (2.1 × 100 mm, 1.7 μm) with mobile phase A (25 mM ammonium acetate/ammonium hydroxide) and B (acetonitrile). Gradient: 85% B (0-1 min) → 65% B (1-12 min, linear) → 40% B (12-16 min) → 85% B (16.1-21.1 min) at 0.3 mL/min.

RPLC mode: An ACQUITY UPLC HSS T3 column (2.1 × 100 mm, 1.8 μm) with mobile phase A (0.1% formic acid or 0.5 mM ammonium fluoride for positive/negative mode) and B (0.1% formic acid in acetonitrile or pure acetonitrile). Gradient: 1% B (0-1.5 min) → 99% B (1.5-13 min) → 1% B (16.6-20 min) at 0.3 mL/min.

Samples (2 μL) were injected in randomized order with column temperature maintained at 25°C. MS detection utilized electrospray ionization (ESI) with the following parameters: Gas1/Gas2 60 psi, curtain gas 30 psi, source temperature 600°C, ion spray voltage ±5500 V. Full-scan MS (m/z 60-1000, 0.20 s/spectrum) and IDA-MS/MS (m/z 25-1000, 0.05 s/spectrum) were acquired in high sensitivity mode with collision energy 35 ±15 eV and declustering potential ±60 V.

(3) Data Processing

Raw data were converted to mzXML format using ProteoWizard MSConvert and processed through XCMS (R package) [13] with centWave algorithm (mass tolerance 10 ppm, peakwidth 10-60 s). CAMERA was employed for isotope/adduct annotation. Metabolites were identified using an in-house database with mass accuracy < 10 ppm and MS/MS spectral matching.

(4) Statistical Analysis

Multivariate analysis included Pareto-scaled PCA and OPLS-DA (ropls package) [14]. Significant metabolites were selected by VIP >1 and *p* <0.05 (Student's *t*-test with Benjamini-Hochberg correction). Hierarchical clustering (Euclidean distance, Ward's method) was performed for expression pattern analysis. Differential metabolites were mapped to KEGG pathways. Pathway-level changes were quantified using Differential Abundance (DA) scores, calculated as the mean fold-change of metabolites within each pathway. Results were visualized by pathway hierarchy classification. Shanghai APTBIO Technology Co., Ltd. supported the sequencing work.

***RNA Sequencing and bioinformatics***

Differential gene expression analysis was conducted using the DESeq2 package [15] to screen for differentially expressed genes (fold change > 2, FDR < 0.05). Gene Ontology (GO), Kyoto Encyclopedia of Genes and Genomes (KEGG), and Gene Set Enrichment Analysis (GSEA) enrichment analyses were performed using the ClusterProfiler [16] and GSEABase [17] packages. The Gene Expression Omnibus (GEO) accession number was GSE246122. The version of R is R 4.3.0 and RStudio is 2023.03.1+446.

**References in the Supplementary Materials**

1. Lagkouvardos, I., et al., *Sequence and cultivation study of Muribaculaceae reveals novel species, host preference, and functional potential of this yet undescribed family.* Microbiome, 2019. **7**(1): p. 28.

2. Lagkouvardos, I., et al., *The Mouse Intestinal Bacterial Collection (miBC) provides host-specific insight into cultured diversity and functional potential of the gut microbiota.* Nat Microbiol, 2016. **1**(10): p. 16131.

3. Niu, M., et al., *Inhibition of heat shock protein (HSP) 90 reverses signal transducer and activator of transcription (STAT) 3-mediated muscle wasting in cancer cachexia mice.* Br J Pharmacol, 2021. **178**(22): p. 4485-4500.

4. Lin, K., et al., *Disrupted methionine cycle triggers muscle atrophy in cancer cachexia through epigenetic regulation of REDD1.* Cell Metab, 2025. **37**(2): p. 460-476.e8.

5. Wei, L., et al., *2-Deoxy-D-glucose Alleviates Cancer Cachexia-Induced Muscle Wasting by Enhancing Ketone Metabolism and Inhibiting the Cori Cycle.* Cells, 2022. **11**(19).

6. Niu, M., et al., *An integrative transcriptome study reveals Ddit4/Redd1 as a key regulator of cancer cachexia in rodent models.* Cell Death Dis, 2021. **12**(7): p. 652.

7. Dubois, B., et al., *A detailed workflow to develop QIIME2-formatted reference databases for taxonomic analysis of DNA metabarcoding data.* BMC Genom Data, 2022. **23**(1): p. 53.

8. Magoč, T. and S.L. Salzberg, *FLASH: fast length adjustment of short reads to improve genome assemblies.* Bioinformatics, 2011. **27**(21): p. 2957-63.

9. Rognes, T., et al., *VSEARCH: a versatile open source tool for metagenomics.* PeerJ, 2016. **4**: p. e2584.

10. Quast, C., et al., *The SILVA ribosomal RNA gene database project: improved data processing and web-based tools.* Nucleic Acids Res, 2013. **41**(Database issue): p. D590-6.

11. Wemheuer, F., et al., *Tax4Fun2: prediction of habitat-specific functional profiles and functional redundancy based on 16S rRNA gene sequences.* Environ Microbiome, 2020. **15**(1): p. 11.

12. Zhang, F., et al., *Prolonged Impairment of Short-Chain Fatty Acid and L-Isoleucine Biosynthesis in Gut Microbiome in Patients With COVID-19.* Gastroenterology, 2022. **162**(2): p. 548-561.e4.

13. Domingo-Almenara, X. and G. Siuzdak, *Metabolomics Data Processing Using XCMS.* Methods Mol Biol, 2020. **2104**: p. 11-24.

14. Worley, B. and R. Powers, *PCA as a practical indicator of OPLS-DA model reliability.* Curr Metabolomics, 2016. **4**(2): p. 97-103.

15. Love, M.I., W. Huber, and S. Anders, *Moderated estimation of fold change and dispersion for RNA-seq data with DESeq2.* Genome Biol, 2014. **15**(12): p. 550.

16. Wu, T., et al., *clusterProfiler 4.0: A universal enrichment tool for interpreting omics data.* Innovation (Camb), 2021. **2**(3): p. 100141.

17. Subramanian, A., et al., *Gene set enrichment analysis: a knowledge-based approach for interpreting genome-wide expression profiles.* Proc Natl Acad Sci U S A, 2005. **102**(43): p. 15545-50.
